# Optimized CRISPR-Cas9 system for efficient engineering of ecDNA in cancer cells

**DOI:** 10.1093/nar/gkag005

**Published:** 2026-01-14

**Authors:** Yohei Sugimoto, Takeru Kachi, Yu Watanabe, Mei Kubokawa, Koichi Ogami, Masaki Kawamata, Seiko Yoshino, Hiroshi I Suzuki

**Affiliations:** Division of Molecular Oncology, Center for Neurological Diseases and Cancer, Nagoya University Graduate School of Medicine, Nagoya 466-8550, Japan; Division of Molecular Oncology, Center for Neurological Diseases and Cancer, Nagoya University Graduate School of Medicine, Nagoya 466-8550, Japan; Division of Molecular Oncology, Center for Neurological Diseases and Cancer, Nagoya University Graduate School of Medicine, Nagoya 466-8550, Japan; Department of Nephrology, Nagoya University Graduate School of Medicine, Nagoya 466-8550, Japan; Division of Molecular Oncology, Center for Neurological Diseases and Cancer, Nagoya University Graduate School of Medicine, Nagoya 466-8550, Japan; Department of Obstetrics and Gynecology, Nagoya University Graduate School of Medicine, Nagoya 466-8550, Japan; Division of Molecular Oncology, Center for Neurological Diseases and Cancer, Nagoya University Graduate School of Medicine, Nagoya 466-8550, Japan; Division of Organogenesis and Regeneration, Medical Institute of Bioregulation, Kyushu University, Fukuoka 812-8582, Japan; Division of Molecular Oncology, Center for Neurological Diseases and Cancer, Nagoya University Graduate School of Medicine, Nagoya 466-8550, Japan; Division of Molecular Oncology, Center for Neurological Diseases and Cancer, Nagoya University Graduate School of Medicine, Nagoya 466-8550, Japan; Institute for Glyco-core Research, Nagoya University, Nagoya 464-8601, Japan; Center for One Medicine Innovative Translational Research, Nagoya University, Nagoya 464-8601, Japan; Inamori Research Institute for Science, Kyoto 600-8411, Japan

## Abstract

Extrachromosomal DNA (ecDNA) amplification represents an emerging mechanism underlying oncogene amplification, tumor heterogeneity, and drug resistance in cancer. However, the biology of ecDNA remains poorly understood because tools to engineer ecDNAs and precisely monitor their dynamics are limited. In particular, genome engineering strategies have not been established for ecDNA, which exists in tens to hundreds of copies within a single cell. Here, we report a systematic validation of ecDNA editing using standard CRISPR-Cas9 system and optimized CRISPR-Cas9 system with safeguard single-guide RNAs (sgRNAs), in which the addition of cytosine extensions finely reduces excessive Cas9 activity. The conventional CRISPR-Cas9 system induced severe cytotoxicity and markedly reduced ecDNA copy number, together with frequent micronucleus formation. Knock-in efficiency was remarkably low, highlighting an intrinsic difficulty in editing ecDNA. In contrast, the safeguard sgRNA strategy not only alleviated cytotoxicity and ecDNA loss in a cytosine-length–dependent manner but also enabled efficient knock-in into multiple ecDNA per cell. Computational simulations suggested that the degree and temporal patterns of multiple DNA cleavage events shape cell death, micronucleus formation, and rapid expansion of knock-in ecDNA. Collectively, optimization of Cas9 activity using safeguard sgRNAs enables efficient and nondisruptive ecDNA engineering, providing a powerful tool to study ecDNA biology.

## Graphical Abstract

**Graphical Abstract:**
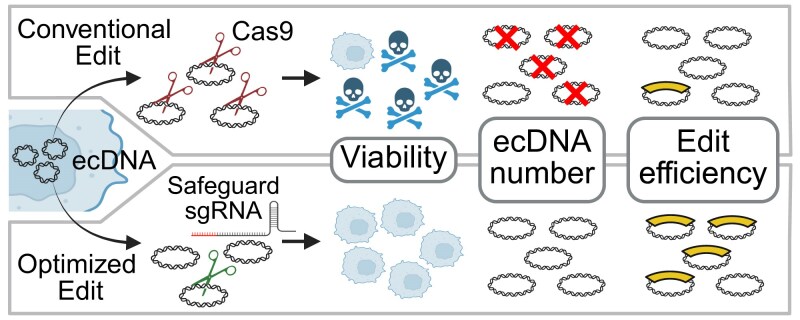

## Introduction

Extrachromosomal DNA (ecDNA) has recently emerged as a key mechanism of oncogene amplification in cancer [1–5]. ecDNAs segregate unevenly during mitosis, leading to heterogeneous oncogene copy numbers among cancer cell populations [1, 6, 7]. This directly induces intratumor heterogeneity and modulates therapeutic response and resistance [1, 2, 7–10]. Historically, ecDNA was described as double minutes (DMs), but their biological and clinical significance remained unclear for decades [11–14]. Recent advances in whole-genome sequencing technologies and bioinformatics have redefined the importance of ecDNA in tumor biology. Clinically, ecDNA has been identified in 10%–60% of various tumor types and is associated with shorter overall survival [1–5]. Several studies have reported that ecDNA exhibits unique intracellular behaviors, such as the formation of ecDNA hubs, which contribute to higher transcription of oncogenes than the same sequences located at their endogenous chromosomal loci [15–17]. However, the molecular mechanisms that enable such dynamic behavior and its functional consequences in cancer remain poorly understood.

Although DNA FISH is widely applicable for investigating the dynamics of ecDNA, molecular tools to manipulate endogenous ecDNAs and probe their functions in living cells remain limited. Recent studies have reported several approaches for live-cell imaging of ecDNA. The first involves dCas9-based systems, called ecTag, in which catalytically inactive Cas9 (dCas9) is guided to ecDNA loci for fluorescent labeling [6]. While this approach is sequence-specific and noninvasive, it fails to reliably detect ecDNAs during mitosis due to signal instability. The second strategy inserts a tetracycline-operator (TetO) array into ecDNAs using CRISPR-Cas9, followed by visualization with fluorescently tagged tetracycline repressor (TetR) proteins [7, 15]. However, because ecDNA exists in high copy numbers—typically dozens per cell—cutting it with Cas9 may cause substantial genomic disruption and unintended cellular consequences. This contrasts with genome editing methods developed for diploid chromosomal loci. Indeed, one earlier study showed that introducing double-strand breaks (DSBs) in ecDNAs with CRISPR-Cas9 induced their aggregation in the nucleus, which subsequently led to micronucleus formation and eventually resulted in its expulsion into the cytoplasm [14, 18]. This finding indicates that cutting ecDNA with Cas9 can strongly compromise its stability and underscores the need for genome editing strategies specifically optimized for multicopy ecDNA.

CRISPR-Cas9 activity is known to cause various adverse effects, including off-target cleavage, chromosomal abnormalities, and reduced cell viability [19–25]. The safeguard single-guide RNA (sgRNA) strategy attenuates Cas9 activity by attaching a variable number of cytosine [C] residues to the 5′ end of the sgRNA [26]. This structural modification gradually decreases sgRNA activity in a cytosine-length–dependent manner, resulting in more controlled DNA cleavage [26]. Consequently, it facilitates genome editing with lower cytotoxicity and improved precision [26].

In this study, we systematically compared a conventional CRISPR-Cas9 system with an optimized CRISPR-Cas9 system employing the safeguard sgRNAs from the viewpoint of various Cas9-induced outcomes: cytotoxicity, maintenance of ecDNA copy number, micronucleus formation, small insertion-deletion (indel) efficiency, and knock-in efficiency via homology-directed repair (HDR). We found that applying a conventional CRISPR-Cas9 system to ecDNA induced strong cytotoxicity and selected cells with low ecDNA copies. In the surviving cells, micronucleus formation was frequently observed after ecDNA cleavage, indicating that remaining damaged ecDNA fragments can be expelled from the nucleus. Furthermore, in the surviving cells, small indel induction frequency and knock-in efficiency were remarkably low. These results highlighted a previously overlooked intrinsic difficulty in editing ecDNA. To overcome these limitations, we applied the safeguard sgRNA system. This approach improved cell viability and preserved ecDNA copy number, enabling more efficient and controlled genome editing of ecDNA. We also developed new computational frameworks to understand how the degree and temporal patterns of multiple DNA cleavage events at multicopy ecDNA shape these cellular consequences.

## Materials and methods

### Cell culture

The human large cell lung carcinoma cell line CORL23 was obtained from the ECACC. Cells were cultured in RPMI-1640 medium (FUJIFILM Wako) supplemented with 10% fetal bovine serum (FBS), 100 U/ml penicillin, and 100 µg/ml streptomycin (Thermo Fisher Scientific). HEK293T cells were cultured in Dulbecco’s modified Eagle’s medium medium (Thermo Fisher Scientific) supplemented with 10% FBS, 2 mM L-glutamine (Thermo Fisher Scientific), 100 U/ml penicillin, and 100 µg/ml streptomycin. ecDNA in CORL23 cells was analyzed using genomic DNA sequencing followed by bioinformatics analysis with AmpliconArchitect (v1.5.r2) [27] and AmpliconClassifier (v1.3.3) [28] under default parameters. Details of these analyses will be described elsewhere.

### DNA FISH on Carnoy-fixed samples

For mitotic cell enrichment, colcemid (Chromosome Science Labo, MA1-05) was added into the culture medium at a final concentration of 0.02 µg/ml for 6–12 h. Cells were treated with Hypotonic Solution II Buffered KCl (Chromosome Science Labo, HS2-50) for 20 min at 37°C, then fixed with Carnoy’s solution (methanol:acetic acid, 3:1) for 10 min at 4°C. Chromosome spreads were prepared by dropping the cells onto humidified glass slides and air-dried. After DNA denaturation for 5 min at 70°C, DNA FISH probes (Chromosome Science Labo) were applied and hybridized overnight at 37°C. The *MYC* probe was labeled with Cy3, and a chromosome 8 probe, used as a control marker for *MYC* localization, was labeled with FITC. In some experiments, the TetO probe labeled with FITC was used instead. Nuclei were counterstained with DAPI and mounted with SlowFade™ Diamond Antifade Mountant (Thermo Fisher Scientific, S36963).

### DNA FISH on PFA-fixed samples

Chamber slides were coated with poly-L-lysine (Sigma–Aldrich, P4707). Cells were fixed with 4% paraformaldehyde (PFA; Nacalai Tesque, 09154) for 10 min and permeabilized with 0.25% Triton X-100 in phosphate buffered saline (PBS) (−) for 5 min at room temperature. Slides were treated with 0.01% pepsin (Sigma–Aldrich, 24-0940) in 0.01 M HCl for 5 min at 37°C and then air-dried. DNA FISH probes were applied, and DNA was denatured for 5 min at 80°C. Hybridization, DAPI counterstaining, and mounting were performed as described earlier.

### Plasmid construction

For CRISPR-Cas9–mediated gene editing, we used the all-in-one vector pS002, which expresses both Cas9 and the sgRNA [29]. The vector was digested with BbsI, and annealed oligonucleotides (listed in Supplementary Table S1) were ligated into the digested sites. The sgRNA targeting the region between *MYC* and *PVT1* (sgMYC) corresponded to the sequence previously used for TetO insertion [7]. To assess the effect of promoter strength, we replaced the CAG promoter driving Cas9 in pS002 with the PGK promoter and compared the performance of CAG and PGK promoters. The HDR repair template was generated by combining a 96-mer TetO fragment excised from the pSP2-96-merTetO-EFS-BLaR plasmid (Addgene, #118713) via restriction enzyme digestion with 500-nucleotide homology arms flanking the Cas9 cleavage site, which were obtained by polymerase chain reaction (PCR) amplification and incorporated into the template. To promote efficient homologous recombination (HR), the HDR repair template plasmid was designed for in-cell linearization by embedding tia1l sgRNA recognition sequences on both sides of the homology arms and integrating a tia1l sgRNA expression cassette within the plasmid [30].

### Transfection

Cells were seeded in six-well plates. Transfection was performed the following day using Lipofectamine 3000 (Thermo Fisher Scientific) according to the manufacturer’s instructions. A total of 2 µg of plasmid DNA was used per well. In some experiments, only the all-in-one vector was introduced, whereas in others, the HDR repair template vector and the all-in-one expression vector were co-transfected at a 1:1 ratio. To prepare the DNA–lipid complex, 1.5 µl of Lipofectamine 3000 was diluted in 100 µl of Opti-MEM. In a separate tube, 2 µg of plasmid DNA together with 4 µl of P3000 reagent was diluted in 100 µl of Opti-MEM. The two mixtures were combined, incubated for 15 min at room temperature, and then added to the cells. After 24 h, the medium was replaced with puromycin-containing medium (3 µg/ml for the sgRNA expression vector alone in CORL23, 3 µg/ml for the sgRNA expression vector alone in HEK293T, or 1 µg/ml for co-transfection with the HDR repair template plasmid; InvivoGen). Puromycin selection was carried out for 2 days. For blasticidin S selection, cells were cultured with blasticidin S (FUJIFILM wako) at a concentration of 40 µg/ml. Viable cells were stained with trypan blue and counted 1 day after puromycin release in Fig. 2B, and 5 days after puromycin release in Fig. 7B. The ecTag analysis was performed as previously described [6].

### Western blot analysis

Western blotting was performed according to standard protocols using primary antibodies against FLAG (Sigma–Aldrich, F1804) and α-tubulin (Sigma–Aldrich, T9026), along with HRP-conjugated secondary antibodies.

### RT-qPCR

Total RNA was extracted using TRIzol Reagent (Thermo Fisher Scientific) and purified with the Direct-zol RNA Miniprep Kit (Zymo Research, R2050) according to the manufacturer’s instructions. Complementary DNA was synthesized from 500 ng of total RNA using SuperScript IV Reverse Transcriptase (Thermo Fisher Scientific) following the manufacturer’s protocol. RT-qPCR analysis was conducted in accordance with the MIQE guidelines. RT-qPCR was performed on a QuantStudio 3 Real-Time PCR System (Thermo Fisher Scientific) with PowerUp SYBR Green Master Mix (Thermo Fisher Scientific). Primer sequences are listed in Supplementary Table S1. Expression of each sgRNA was quantified using a single primer set targeting a common sequence shared among all sgMYC sgRNAs (Supplementary Fig. S2B). The PCR cycling conditions were as follows: 95°C for 2 min, followed by 40 cycles of 95°C for 1 s and 60°C for 30 s. Amplification specificity was confirmed by melting curve analysis. Expression levels were normalized to GAPDH expression as an internal control, and relative expression levels were calculated using the ΔCt method. Experiments were performed with technical triplicates.

### ecDNA copy number analysis

To quantify ecDNA copy number, metaphase DNA FISH images of Carnoy’s fixed spreads were acquired using a BZ-X810 microscope (KEYENCE). FISH signals were analyzed with the metaseg, meta_overlay, and stat_fish functions of ecSeg (https://github.com/UCRajkumar/ecSeg) [31, 32]. The metaseg module segments metaphase images based on DAPI morphology and classifies DNA regions into chromosomal or extrachromosomal compartments. DNA FISH signals are then assigned to these compartments with meta_overlay, enabling automated detection and classification of signals. For interphase FISH images, approximate ecDNA copy number per cell was quantified using the stat_fish function.

### Flow cytometry and cell sorting

Because puromycin selection may mask Cas9-induced cytotoxicity, cells were harvested 24 h after transfection without puromycin treatment and subjected to flow cytometric analysis. Apoptotic and necrotic cells were assessed using the Apoptosis Detection Kit (Nacalai Tesque, 15342-54) according to the manufacturer’s instructions. Data acquisition and cell sorting were performed on a BD FACSAria Fusion (BD Biosciences). Cells positive for Annexin V and/or propidium iodide (PI), as well as double-negative cells, were sorted into two different populations. Only Annexin V and/or PI-positive cells were subsequently fixed with Carnoy’s solution and subjected to DNA FISH analysis as described above. Flow cytometric data were analyzed using FlowJo software v10 (BD Biosciences).

### T7E1 assays and TIDE analysis

Cells were transfected and treated with puromycin for 2 days as described earlier. Genomic DNA (gDNA) was then extracted with the Monarch Spin gDNA Extraction Kit (NEB, T3010S). T7E1 assays were performed as previously described [26]. Briefly, Cas9 target sites were amplified by PCR using PrimeSTAR GXL (Takara, R050A), followed by denaturation and reannealing of the PCR products. T7 endonuclease I was then added to cleave the mismatched DNA. Reaction products were subsequently analyzed by agarose gel electrophoresis. Gel images were quantified using Fiji (ImageJ). Primer sequences are listed in Supplementary Table S1. Tracking of indels by decomposition (TIDE) analysis (https://tide.nki.nl) was performed using Sanger sequencing traces generated from target-specific PCR products by Eurofins [33]. Default parameters were applied. The analysis window was set to −10 to +10 bp around the Cas9 cleavage site to avoid a downstream region of the sgMYC target site on the ecDNA, which contains a T-rich repeat sequence that interferes with primer design for PCR amplification and subsequent Sanger sequencing.

### Imaging of micronuclei

Cells were transfected and fixed on the second day of puromycin selection. DNA FISH on PFA-fixed samples was then performed according to the procedure described earlier. Annexin V staining was performed using the Apoptotic, Necrotic & Healthy Cells Quantification Kit (Biotium, 30 018). Micronuclei were imaged using a Nikon AX-R confocal microscope equipped with a CFI Plan Apochromat Lambda D 100 × oil immersion lens and NIS-Elements software (Nikon). Images in Fig. 4A and C were acquired as Z-stacks and deconvolved using NIS-Elements software. Supplementary Fig. S3A shows single-plane images acquired without deconvolution.

### Cell line generation for ecDNA imaging and ecDNA detection by TetR-EGFP

CORL23 cells were first infected with a lenti F9-TetR-EGFP-IRES-HygroR lentiviral vector, which was generated by replacing the puromycin resistance gene of the original lenti F9-TetR-EGFP-IRES-PuroR (Addgene, #117049) with a hygromycin resistance gene. Hygromycin (FUJIFILM Wako) selection at a final concentration of 300 µg/ml was initiated the day after infection and maintained for 7 days to establish stable cell lines expressing TetR-EGFP. Genome editing was subsequently performed according to the procedure described in the Transfection section. Successfully edited clones were maintained under continuous selection with both blasticidin S and hygromycin. ecDNA was visualized by culturing cells on poly-L-lysine–coated dishes, fixing with 4% PFA for 5 min, and staining with Hoechst. Images were acquired as Z-stacks using a Nikon AX-R confocal microscope.

### Computational framework for ecDNA DSB modeling

In this study, we set up new simulations in which Cas9 binding and DSBs were randomly distributed across the defined time frame, and the temporal patterns of Cas9-induced DSBs on multicopy ecDNA were recorded for each cell with various ecDNA copy numbers (Fig. 5A). Overview of this computational framework is summarized in Supplementary Fig. S4.

(i) Computational simulation of temporal patterns of ecDNA DSBs

First, in the initial simulation setup, for each ecDNA copy, we randomly placed a predetermined number of Cas9 binding and DSB events across 10 000 time frames. The frequency (f) of Cas9 binding and DSB events was varied across simulations, spanning values from 1 to 10 000 events. Within these 10 000 time frames, for each ecDNA copy, the first binding event was recorded as a DSB event. Next, we designated the first 100 time frames out of the 10 000 as the time window for data analysis (“sampling window”), and we recorded the number of DSBs in each time frame as well as the total number of DSBs (“total DSB”) within this sampling window. In addition, among the DSB counts per time frame, the maximum value was recorded as the “max DSB number.” We tested copy numbers of 2, 10, 20, 30, 40, 50, 60, 70, 80, 90, and 100 and analyzed 1000 cells in each condition. For simplicity, we did not consider potential competition among multiple ecDNA copies. The results of initial simulations are summarized in Fig. 5 and Supplementary Figs S5 and S6.

(ii) Inference of Cas9-induced cytotoxicity

Based on previous observations in which the number of target sites (0–20 copies) correlates well with antiproliferative effect of Cas9-mediated DSBs in CRISPR–Cas9 essentiality screens [34–36], we set the cell survival probability P as follows:


(1) \begin{eqnarray*}
\begin{array}{@{}*{1}{c}@{}} {P\left( {cp1} \right) = \left\{ {\begin{array}{@{}*{1}{c}@{}} {1 - \frac{{cp1}}{{Mt}}\ \left( {0 \le cp1 \le Mt} \right)}\\ {0\ \left( {\mathrm{otherwise}} \right)} \end{array}} \right.} \end{array},
\end{eqnarray*}

where cp1 and Mt are total DSB number and lethal threshold, respectively (Fig. 5E).

We converted the ecDNA copy number distribution in CORL23 cells in Fig. 2D into a frequency table with bin widths of 10 and defined the initial population by multiplying the frequency table from untreated cells by 1000 (“initial CORL23 population”). For such an initial population, we simulated changes in the cell population under a range of Cas9 binding frequencies (f) and lethal thresholds and compared the results with the experimental outcomes for [0C] sgRNA (standard sgRNA). We used the sum of squared residuals (SSR) as the error function, calculated as follows: SSR = ∑(Experimental data – Simulated data)^2^; we determined stable lethal threshold [33 copies for high-frequency settings (Fig. 5F, see the dashed boxes)]. Using this threshold, we determined the Cas9 binding frequencies that best fit the experimental data for the [0C], [5C], [10C], [15C], and [20C] sgRNA conditions. The corresponding frequencies for [0C], [5C], [10C], [15C], and [20C] sgRNAs were 770, 170, 40, 30, and 3, respectively, and this mapping was used consistently in all subsequent analyses. The results are summarized in Fig. 5 and Supplementary Figs S7 and S8.

(iii) Temporal patterns of multiple DSBs in the surviving cells

We computationally analyzed the degree and temporal pattern of multiple DSBs on multicopy ecDNAs in the surviving cells, which originate from initial CORL23 population. To assess whether multiple DSBs accumulate within short time intervals, we analyzed the number and temporal patterns of DSBs within windows of five time frames. Specifically, we examined how multiple DSBs clustered within each five-time-frame window and whether individual DSBs were temporally associated with other DSB events. In the micronuclei analysis, we determined the optimal threshold by calculating the correlation and SSR between the fraction of micronuclei-positive cells and the fraction of cells exhibiting clustered multiple DSBs (Supplementary Fig. S10). The results are summarized in Fig. 6 and Supplementary Figs S9–S11.

(iv) Uneven segregation of ecDNA and its impacts on knock-in efficiency

In the knock-in simulation, the initial CORL23 cell population was subjected to total DSB-dependent cellular selection under various Cas9 binding frequencies, and 20.6% of temporally isolated single DSBs were further converted into TetO ecDNA. From this population, 10 000 cells were randomly sampled. For each cell, ecDNA copies with and without TetO knock-in were amplified to 2n copies, and each ecDNA was segregated into two daughter cells according to a binomial trial B(2n, 0.5). These procedures were based on previous studies [1, 7]. Subsequently, we applied the following selection pressures depending on the copy number of TetO knock-in ecDNA:


(2) \begin{eqnarray*}
\begin{array}{@{}*{1}{c}@{}} {P\left( {cp2} \right) = \left\{ {\begin{array}{@{}*{1}{c}@{}} {0.45 + \frac{{cp2}}{{30}}\ \left( {0 \le cp2 \le 6} \right)}\\ {0.6 + \frac{{cp2}}{{120}}\ (6 < cp2 \le 30)}\\ {0.85\ (30 < cp2)} \end{array}} \right.}, \end{array}
\end{eqnarray*}

where cp2 is the copy number of TetO knock-in ecDNA (Fig. 9C (ii)).

This selection pressure was defined by comparing the ecDNA copy number distribution patterns in the presence versus absence of blasticidin S and estimating the inflection point under blasticidin S treatment (Supplementary Fig. S14). However, as shown in Supplementary Fig. S15, essentially the same overall trend was observed even when different selection pressure settings were applied. We also performed simulations that incorporated the intrinsic selective pressure of Myc ecDNA itself as a second-stage selection pressure, but the results were comparable (data not shown). We randomly sampled 10 000 cells from the surviving population and then iteratively applied the above cycle of unequal segregation and selection. This cycle was repeated 10 times, and for each parameter setting, the entire simulation was run 50 independent times to confirm the high reproducibility of the results. The results are summarized in Fig. 9 and Supplementary Figs S14 and S15.

### Statistical analysis

**Figure 1.**
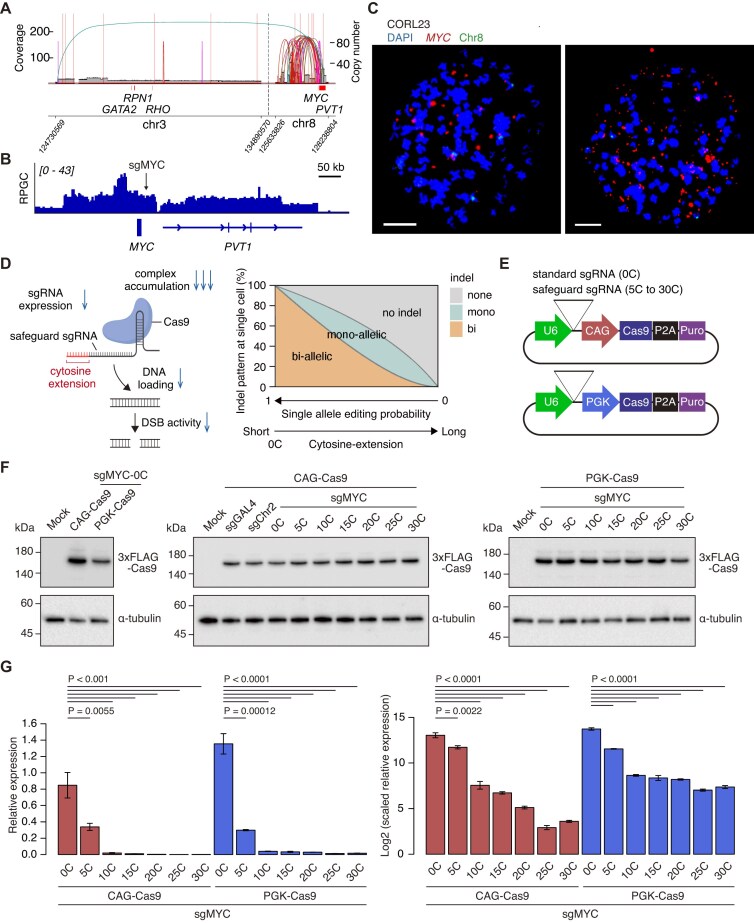
Application of the safeguard sgRNA system in ecDNA-positive CORL23 cells. (**A**) AmpliconArchitect analysis showing the presence of ecDNA structures involving the *MYC* and *PVT1* loci in CORL23 cells.(**B**) IGV snapshot showing genome sequence profiles at the *MYC* and *PVT1* loci. (**C**) Representative FISH images of CORL23 cells using an *MYC* locus probe (red) together with a chromosome 8 probe (green; control for the *MYC*-containing chromosome). Two representative single cells with different *MYC* ecDNA copy numbers are shown. Scale bar, 10 µm. (**D**) Schematic illustration of the proposed molecular mechanism by which safeguard sgRNAs fine-tune Cas9 activity. Cytosine extensions decrease Cas9 activity and attenuate Cas9-mediated DNA cleavage via multiple mechanisms, including reduced sgRNA synthesis and impaired target loading efficiency of Cas9–sgRNA RNP complexes (left). When using potent sgRNAs, [C]-extended sgRNAs exhibited decreased bi-allelic indels on chromosomal targets and increased mono-allelic indels in a length-dependent manner (right). This figure was adapted from Kawamata *et al*. [26], licensed under the CC BY 4.0. (**E**) Schematic of all-in-one CRISPR plasmids expressing standard and safeguard sgRNAs. (**F**) Western blot analysis of FLAG-tagged Cas9 proteins expressed from the indicated all-in-one plasmids in CORL23 cells. (**G**) Quantification of sgRNA expression levels by RT-qPCR. Left, normalized expression values; right, normalized values multiplied by 1000 and log_2_-transformed. Data represent mean ± SD for *n* = 3 technical replicates. Statistical significance was assessed using a two-sided Student’s *t*-test.

**Figure 2.**
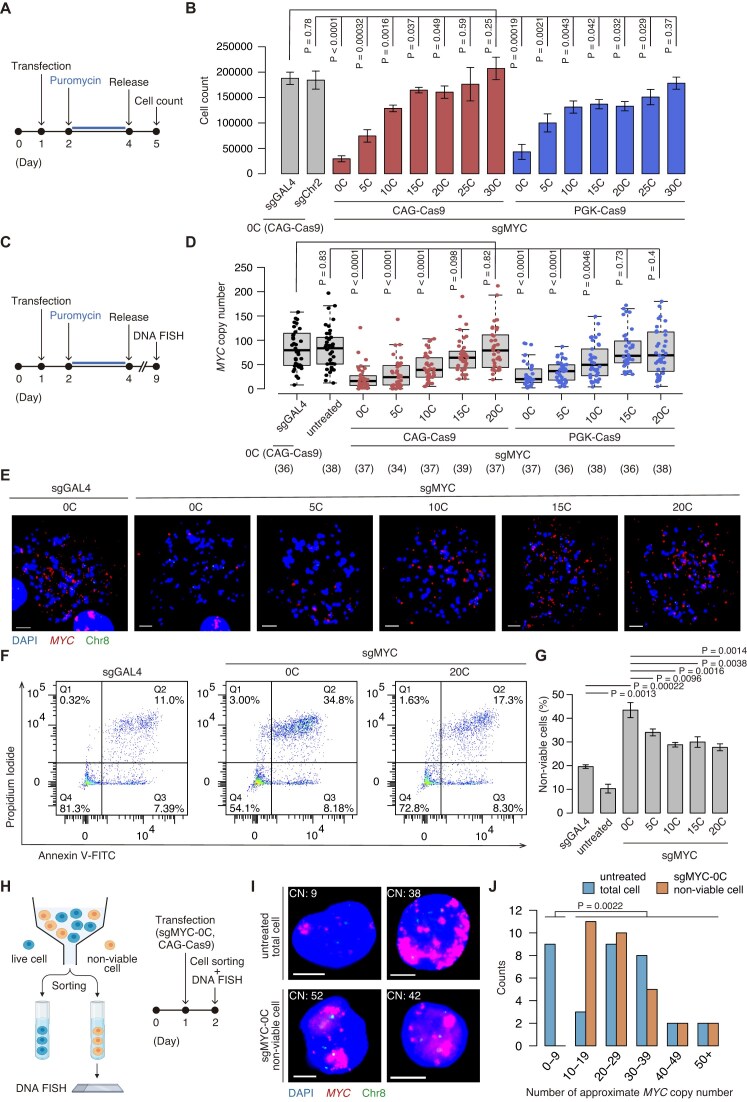
Effects of standard and safeguard sgRNAs on cell viability and ecDNA copy number. (**A**) Experimental scheme for assessing cell survival in panel (B). (**B**) Quantification of CORL23 cell numbers after expression of all-in-one plasmids with the indicated sgRNAs. sgGAL4 and sgChr2 are [0C] sgRNAs. Data represent mean ± SD for *n* = 3 biological replicates. Statistical significance was assessed using a two-sided Student’s *t*-test. (**C**) Experimental scheme for DNA FISH analysis in panel (D). (**D**) Box-and-whisker plots showing *MYC* ecDNA copy numbers measured from DNA FISH images in cells expressing all-in-one plasmids with the indicated sgRNAs. Sample sizes (*n*) are shown below sgRNA labels. Statistical significance was assessed using a Wilcoxon rank-sum test. (**E**) Representative DNA FISH images. Scale bar, 10 µm. (**F**) Cells expressing all-in-one plasmids with the indicated sgRNAs were stained with Annexin V-FITC and PI, then analyzed by flow cytometry. (**G**) The percentage of non-viable cells, defined as the sum of Annexin V+/PI− (Q3), Annexin V+/PI+ (Q2), and Annexin V−/PI+ (Q1) populations in panel (F), was determined. Data represent the mean ± SD from *n* = 3 biological replicates. Statistical significance was assessed using a two-sided Student’s *t*-test. (**H**) Experimental scheme for flow cytometry-based cell sorting and subsequent DNA FISH analysis in panels (I) and (J). (**I**) Representative DNA FISH images. Scale bar, 10 µm. CN indicates approximate ecDNA copy number. (**J**) Quantification of MYC ecDNA foci in untreated total cells and sgMYC-0C–expressing non-viable cells. Statistical significance was determined using Fisher’s exact test.

In Figs 1G, 2B, 2G, 4B, 4D, 7B, and 10E, statistical significance was evaluated using two-sided Student’s *t*-tests between the two indicated groups. In Figs 2D, 7D–F, 10B, and 10C, and Supplementary Fig. S12B–D, statistical significance was evaluated using Wilcoxon rank-sum tests between the two indicated groups. In Fig. 2J, statistical significance was evaluated using Fisher’s exact test (comparison between untreated total cells and sgMYC-0C non-viable cells for the proportion of cells with 0–9 versus >10 ecDNA foci). In Figs 5H, 5J, 5K, 6F, 6J, and 9B, and Supplementary Fig. S7C, linear regression curves, Pearson’s correlation coefficients (r) with 95% confidence intervals, and *P* values were evaluated.

## Results

### Application of conventional and safeguard sgRNA strategies in an ecDNA-positive cancer cell line

In this study, we used the lung cancer cell line CORL23, which was identified through screening of ecDNA-positive cancer cell lines (data not shown) (Fig. 1A–C). Based on genome sequencing data, CORL23 cells carry ecDNAs containing the *MYC* and *PVT1* loci (Fig. 1A and B). In metaphase DNA FISH images, extrachromosomal amplification of the *MYC* locus was confirmed, showing variable degrees of amplification among individual cells with a median copy number of 86 (Fig. 1C). This finding is consistent with the characteristic copy number heterogeneity and high-level amplification of ecDNA [1, 2, 5–7].

To visualize ecDNA in CORL23 cells, we first applied the previously reported ecTag system [6]. The ecTag system utilizes the Casilio platform to recruit multiple fluorescent protein molecules to a prespecified sgRNA target locus using three components: sgRNAs with Pumilio/FBF (PUF) RNA binding sites (sgRNA-PBS), catalytically inactive Cas9 (dCas9), and a Clover-PUF fusion fluorescent protein (Clover-PUFa) [37]. To enhance the efficiency of ecDNA detection, we generated an all-in-one ecTag vector incorporating all three components into a single construct (Supplementary Fig. S1A). However, the efficiency and specificity of ecDNA detection were low (Supplementary Fig. S1B and C). Because both targeting efficiency and on-target efficiency of the ecTag system varied substantially across distinct target sequences [6], we next attempted to directly manipulate endogenous ecDNA by inserting a TetO repeat sequence for visualization with fluorescently tagged TetR proteins [7, 15]. During an initial attempt to knock-in the TetO repeat sequence using the standard CRISPR-Cas9 system (Supplementary Fig. S2A, [0C]), we found that isolating hundreds of single-cell clones was necessary to obtain a few successful clones containing multiple TetO repeat–positive ecDNAs (data not shown). Given a previous report showing that Cas9-mediated DSBs in ecDNA induced its aggregation in the nucleus and ultimately resulted in its expulsion into the cytoplasm [18], we hypothesized that the consequences and efficiency of CRISPR-Cas9 engineering differ substantially between chromosomal and ecDNA targets.

To investigate this, we systematically analyzed the outcomes of the CRISPR-Cas9 system with standard sgRNAs and safeguard sgRNAs targeting the *MYC* locus. We previously demonstrated that the addition of cytosine stretches to the 5′ end of conventional sgRNAs (i.e. safeguard sgRNAs) results in length-dependent inhibition of functional Cas9 complex formation [26]. Short cytosine extensions reduced p53 activation and cytotoxicity in human induced pluripotent stem cells (iPSCs) and enhanced HDR while maintaining Cas9 activity and biallelic editing [26]. Longer extensions further decreased on-target activity but improved the specificity and precision of monoallelic editing [26]. This modulation of Cas9 activity arises from multiple mechanisms, including reduced transcriptional efficiency of cytosine-extended sgRNAs and impaired target loading efficiency of Cas9–sgRNA ribonucleoprotein (RNP) complexes (Fig. 1D) [26]. Safeguard sgRNAs aid in controlling bi-allelic versus mono-allelic editing of chromosomal targets (Fig. 1D). We first generated all-in-one plasmids expressing Cas9, a puromycin-resistance cassette, and sgRNAs with cytosine extensions of varying length ([C] = 0, 5, 10, 15, 20, 25, and 30 nucleotides) at the 5′ end (hereafter denoted as [0C] sgRNA to [30C] sgRNA) (Supplementary Fig. S2A). We also tested the effects of promoter strength on Cas9 expression by preparing two versions of the plasmids with the strong CAG promoter and the moderate or weak PGK promoter (Fig. 1E). We confirmed that Cas9 protein levels depended on promoter strength, but that [C] extensions did not largely affect Cas9 protein levels for each promoter (Fig. 1F). In both promoter contexts, sgRNA expression decreased markedly with increasing [C]-extension length (Fig. 1G and Supplementary Fig. S2B). These results are highly consistent with dramatic reduction of [C]-length–dependent sgRNA expression, which was validated in mouse embryonic cells (mESCs) in our previous report [26]. This reinforces the universality of the safeguard sgRNA approach in diverse cells.

### Standard CRISPR-Cas9 system induces cytotoxicity and ecDNA loss, which are alleviated by safeguard sgRNAs

We next assessed the survival of ecDNA-positive CORL23 cells after expression of the safeguard sgRNAs (Fig. 2A and B) and the maintenance of ecDNA (Fig. 2C–E). We analyzed cells successfully transfected with all-in-one plasmids by selecting them with puromycin treatment (Fig. 2A and C). As a negative control, we included an [0C] sgRNA targeting the yeast gene *GAL4* (sgGAL4) [6]. As a chromosomal control, we analyzed the performance of [0C] sgRNA targeting a control sequence in chromosome 2 (Chr2) (sgChr2) [38] and found no difference compared with sgGAL4, indicating that targeting a chromosomal region does not induce cytotoxicity. Under the CAG promoter, relative to the negative controls sgGAL4 and sgChr2, severe cytotoxicity was induced by a conventional [0C] sgRNA targeting ecDNA (Fig. 2B). Lowering Cas9 expression with the PGK promoter did not alleviate this cytotoxicity, as [0C] sgRNA induced comparable cell death to that observed with the CAG promoter. In both promoter conditions, safeguard sgRNAs progressively reduced this effect, and cell survival increased with longer [C] extensions (Fig. 2B). Furthermore, under the CAG promoter, DNA FISH analysis of the *MYC* locus demonstrated that surviving cells treated with a conventional [0C] sgRNA showed markedly lower ecDNA copy numbers, whereas sgGAL4-expressing cells maintained ecDNA copy number at levels comparable to untreated cells (Fig. 2D and E). Notably, the depletion of cells with high ecDNA copies was reversed stepwise by increasing the number of [C] in the safeguard sgRNAs (Fig. 2D and E). Reducing Cas9 expression with the PGK promoter yielded a comparable decrease in *MYC* ecDNA, and [C] extensions mitigated this effect (Fig. 2D). Together, these results demonstrate that conventional genome editing strategies are poorly suited for ecDNA, whereas the use of safeguard sgRNAs reduces cytotoxicity and maintains ecDNA copy number. Moreover, lowering Cas9 expression by changing the promoter was insufficient to achieve these effects, suggesting that broader regulation of Cas9 activity by safeguard sgRNAs is useful to minimize cytotoxicity and preserve ecDNA.

To investigate the mechanism underlying depletion of cells with high ecDNA copy number in standard CRISPR-Cas9 system, we next assessed cell viability by flow cytometry using Annexin V and PI staining. As shown in Fig. 2F and G, the [0C] sgRNA induced substantial apoptosis at an early time point, 24 h after transfection. The application of safeguard sgRNAs reduced Cas9-induced cytotoxicity in a [C]-length–dependent manner (Fig. 2F and G).

To further examine whether there is a link between Cas9-induced cell death and ecDNA copy number, non-viable cells (Annexin V and/or PI–positive cells) were sorted by flow cytometry and subjected to DNA FISH analysis (Fig. 2H). Approximate ecDNA copy number per interphase cell was quantified using the stat_fish function in the ecSeg toolkit [32]. As shown in Fig. 2I and J, non-viable cells were devoid of cells with low ecDNA copy numbers compared with the total population. These findings indicate that cells harboring high-copy ecDNA are preferentially eliminated by the [0C] sgRNA possibly due to pronounced DSB-induced cytotoxicity.

### Profiling of small indel frequency in chromosomal and ecDNA target sequences

**Figure 3.**
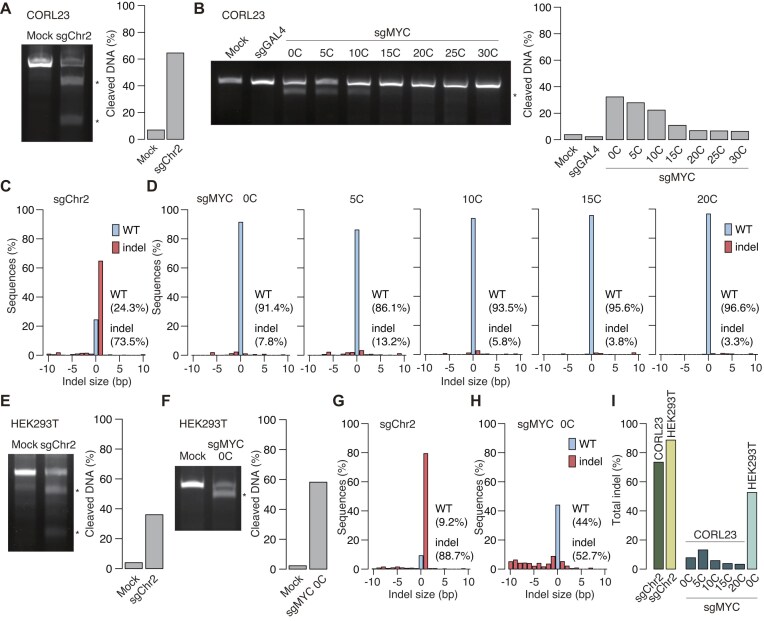
Evaluation of indel formation in ecDNA and chromosomal DNA using T7E1 assay and TIDE analysis. (**A, B**) Agarose gel images (left) of T7E1 assays in CORL23 cells showing indel frequencies at a region on chromosome 2 (**A**) and at the *MYC* locus in ecDNA (**B**). The sgRNAs expressed in each condition are indicated above the gels. Cleaved DNA fragments are marked by asterisks. Quantification (right) shows the proportion of cleaved bands relative to the total DNA signal. In panel (B), the smaller one of the cleaved DNA fragments (∼80 bp) was not detected because of its small size. (**C, D**) TIDE analysis showing the indel frequencies in the range of −10 to +10 bp at a region on chromosome 2 (**C**) and at the *MYC* locus in ecDNA (**D**) using the samples in panels (A) and (B). The sgRNAs expressed in each condition are indicated in the plots. (**E**–**H**) T7E1 assays (**E** and **F**) and TIDE analyses (**G** and **H**) performed in HEK293T cells, as in panels (A–D). (**I**) Comparison of indel frequencies in CORL23 and HEK293T cells. Total indel frequencies for each sgRNA were calculated by summing all indel frequencies from the TIDE analyses shown in panels (C, D, G, and H). All experiments in this figure were performed using all-in-one plasmids with the CAG promoter.

Cas9-mediated DSBs at target loci are primarily repaired by error-prone pathways such as non-homologous end joining (NHEJ) or microhomology-mediated end joining (MMEJ) [39, 40], typically generating small indels at the cleavage site. In the context of ecDNA, whose copy number was markedly reduced by a conventional [0C] sgRNA, the ecDNA editing status in the remaining cells was unclear. To evaluate the editing efficiency of CRISPR-Cas9 on ecDNA, we examined the frequency of indels (Fig. 3A and B). Indels were assessed with the standard T7 endonuclease I (T7E1) assay, which detects DNA fragments carrying mismatches generated during repair of Cas9-induced DSBs. For the Chr2-targeting control sgRNA, 64.5% of DNA was edited in CORL23 cells (Fig. 3A), indicating that chromosomal editing efficiency remains robust in ecDNA-positive cells. By contrast, with ecDNA-targeting sgRNAs, the surviving cells transfected with the *MYC*-targeting [0C] sgRNA exhibited detectable indel formation, but the indel frequency was limited to 32.3%—lower than that observed at the chromosomal site on Chr2 (Fig. 3B). Consistent with the length-dependent inhibition of functional Cas9 complex formation, [C] extensions reduced indel frequency in a length-dependent manner (Fig. 3B).

To obtain a more quantitative view of indel efficiency, we further employed TIDE analysis [33]. This method uses standard Sanger sequencing of PCR amplicons from the target locus, followed by computational analysis of the mixed sequence traces. Due to the presence of a T-rich repeat sequence near the sgMYC target sites, we focused on the frequency of “small” indels with −10 to +10 bp range in this analysis. Consistent with the T7E1 assay, TIDE analysis confirmed high small indel frequency at chromosomal target sequences (Fig. 3C) and low small indel frequency at ecDNA in CORL23 cells (Fig. 3D).

We next investigated whether the low indel frequency of ecDNA is attributable to differences in the chromosomal versus ecDNA configuration of the *MYC* target sequence. To this end, we tested the performance of the same [0C] sgRNAs targeting Chr2 and chromosomal *MYC* in HEK293T cells. T7E1 assays confirmed indel formation at both targets (Fig. 3E and F), and subsequent quantification by TIDE analysis revealed that both targets exhibited high small indel frequencies (Fig. 3G and H). When comparing results from CORL23 and HEK293T cells, the performance of [0C] sgRNAs targeting Chr2 was comparable (Fig. 3I). However, the small indel frequency at the *MYC* locus in ecDNA in CORL23 cells was substantially lower than that in HEK293T cells (Fig. 3I). Taken together, these results suggest that the frequency of small indel formation at ecDNA target may be lower than that at chromosomal target.

**Figure 4.**
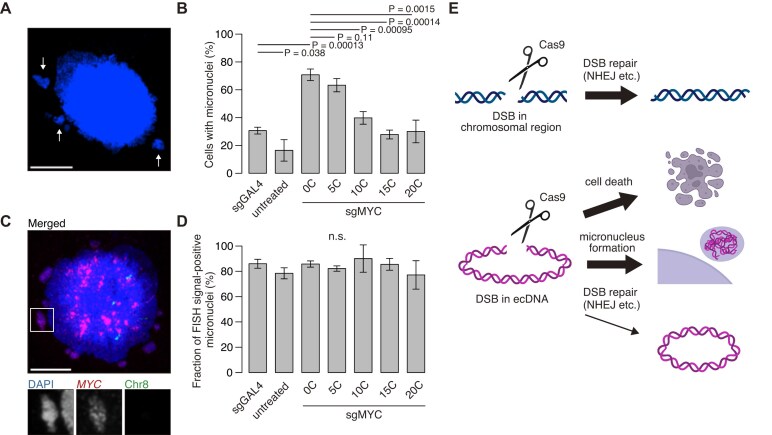
Effects of standard and safeguard sgRNAs on micronucleus formation. (**A**) Representative image of a CORL23 cell containing micronuclei. White arrows indicate micronuclei. Scale bar, 10 µm. (**B**) Proportion of CORL23 cells containing micronuclei after expression of all-in-one CRISPR plasmids with the indicated sgRNAs. Data represent mean ± SD for *n* = 3 biological replicates. The numbers of cells analyzed were 65 (sgGAL4), 61 (untreated), 48 [0C], 54 [5C], 55 [10C], 62 [15C], and 48 [20C]. Statistical significance was assessed using a two-sided Student’s *t*-test. (**C**) Representative DNA FISH image of a CORL23 cell containing ecDNA-positive micronuclei. The enlarged view of the white boxed region is presented below. Scale bar, 10 µm. (**D**) Proportion of ecDNA-positive micronuclei. Data represent mean ± SD for *n* = 3 biological replicates. The numbers of cells analyzed were 41 (sgGAL4), 37 (untreated), 49 [0C], 51 [5C], 38 [10C], 40 [15C], and 41 [20C]. Statistical significance was assessed using a two-sided Student’s *t*-test. All experiments in this figure were performed using all-in-one plasmids with the CAG promoter. (**E**) Schematic illustrating distinct outcomes of CRISPR-based chromosomal and ecDNA targeting.

**Figure 5.**
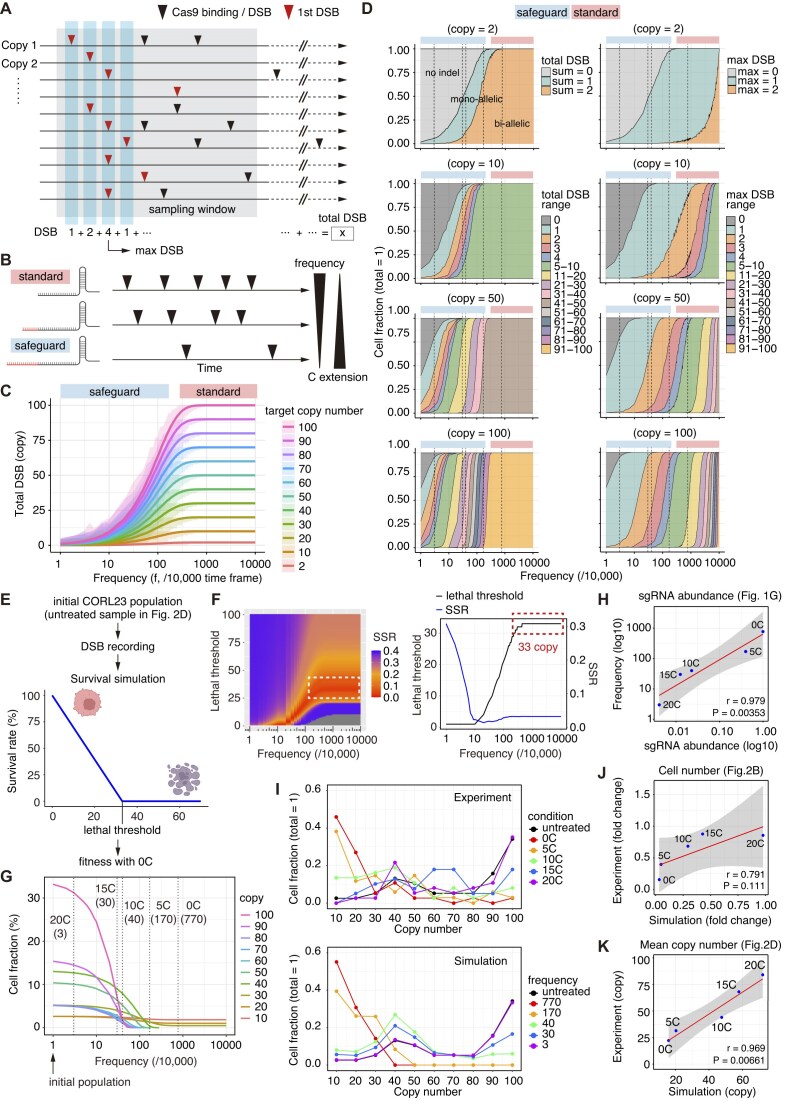
Time-course simulation of Cas9-induced DSBs on multicopy ecDNA. (**A**) Scheme for time-course simulation of Cas9-induced DSBs. (**B**) Effects of safeguard sgRNAs on the frequency of Cas9 binding and DSB. (**C**) Simulations of total amount of DSBs in cells with various numbers of target sites. The shaded area represents the max–min range. (**D**) Cell-level quantification of total amount of DSBs (left) and max DSB number per one time frame (right) across various frequencies of Cas9 binding and DSB events. The dotted lines represent the inferred frequencies of Cas9 binding for each sgRNA, and the corresponding sgRNAs are indicated in panel (G). (**E**) Scheme for total DSB-dependent cytotoxicity. (**F**) Identification of the lethal thresholds that minimized SSR for the results of standard sgRNA ([0C] sgRNA). Left panel shows the relationships among lethal threshold, SSR, and frequencies of Cas9 binding and DSB events. Note that lethal threshold is constant for a wide range of highly efficient sgRNA (lethal threshold = 33, see the dashed boxes). (**G**) Changes in cell fraction in the CORL23 cell population under various frequencies of Cas9 binding. (**H**) Correlation between sgRNA abundance and inferred frequencies of Cas9 binding for each sgRNA. The dotted lines represent the inferred frequencies of Cas9 binding for each sgRNA. (**I**) Comparison of ecDNA copy number distribution in experiments (top) and simulations (bottom). (**J, K**) Correlation between experimental and simulation results of cell number changes (**J**) and mean ecDNA copy number (**K**). In panels (H), (J), and (K), linear regression curves, Pearson’s correlation coefficients (*r*) with 95% confidence intervals, and *P* values are shown.

**Figure 6.**
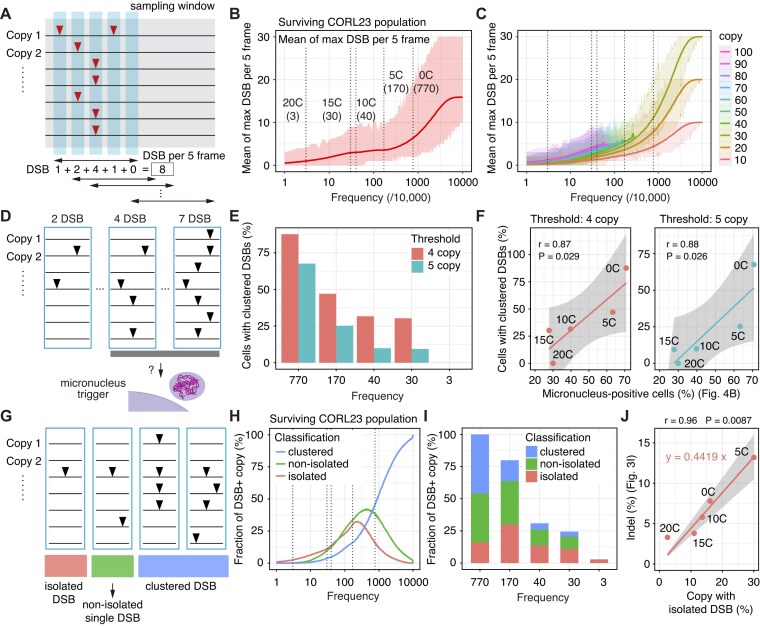
Cas9 binding frequency modulates temporally clustered versus isolated DSBs. (**A**) Scheme for analysis of temporal accumulation of multiple DSBs. (**B**) The trajectory of the maximum DSB count per five time frames in the surviving CORL23 population under various Cas9 binding frequencies in the surviving cells. The shaded area represents the max–min range. (**C**) Replot of panel (B) according to ecDNA copy number per cell. (**D**) Association between clustered DSBs and micronuclei formation. (**E**) Proportion of cells with clustered DSBs under thresholds of four and five copies. (**F**) Correlation between the proportion of micronuclei-positive cells (Fig. 4B) and cells with clustered DSBs (threshold 4: left, 5: right). (**G**) Extraction of temporally isolated DSBs. (**H**) Dynamics of the fractions of copies exhibiting temporally isolated single DSBs, temporally non-isolated single DSBs, or multiple DSBs across varying Cas9 binding frequencies. (**I**) Replot of the subset of panel (H) representing sgRNAs used in the experiments. (**J**) Correlation between the proportion of ecDNA with small indels (Fig. 3I) and the simulated copy fraction with temporally isolated single DSBs. In panels (F) and (J), linear regression curves, Pearson’s correlation coefficients (r) with 95% confidence intervals and *P* values are shown. In panels (B), (C), and (H), the dotted lines represent the inferred frequencies of Cas9 binding for each sgRNA, and the corresponding sgRNAs are indicated in panel (B).

We note that comparisons of indel frequencies between sgRNAs targeting ecDNA and chromosomal loci may be influenced by sequence-dependent differences in sgRNA efficiency. In addition, larger indels (e.g. 10–30 bp range indels and kb-Mb range indels) and/or other complex rearrangements should be evaluated in future studies. The observed low frequency of small indel formation at ecDNA may be associated with ecDNA loss following Cas9-mediated cleavage through micronuclei formation (see later results in Fig. 4) or the shift from small indel formation to larger indel formation and other complex rearrangements through more efficient targeting in the setting of ecDNA (see later simulation results in Figs 5 and 6 and discussion). In the former scenario, indel frequencies measured on the remaining ecDNA may underestimate the true editing efficiency. Supporting the latter scenario, a recent report demonstrated that efficient Cas9-based mutagenesis leads to an increase in the size of small indels [41]. Furthermore, particularly in the experiments with the [0C] and [5C] sgRNAs, because only a very small fraction of cells survived, cells failing to express sufficient Cas9 may be preferentially enriched among the surviving population, leading to ostensibly low editing frequency. Overall, these findings highlight an inherent difficulty in Cas9-mediated editing of ecDNA, which has thus far been overlooked.

### The effects of standard and safeguard sgRNA approaches on micronucleus formation

Although chromosomal DSBs undergo typical DNA repair pathways, such as NHEJ, MMEJ, and HR, a previous study using the ecDNA-positive cell line COLO320DM reported that Cas9-induced DSBs in ecDNA led to nuclear aggregation of ecDNA fragments, which were subsequently released from the nucleus during mitosis and appeared as micronuclei [18]. Therefore, in addition to induction of cell death (Fig. 2), micronuclei formation may contribute to loss of ecDNA (Fig. 2C and D) and to the low small indel frequency of the remaining ecDNA (Fig. 3) in surviving Cas9-treated cells as an alternative consequence of DSBs. We next determined whether similar processes occurred under our experimental conditions. Consistent with that report [18], micronucleus formation was frequently observed in puromycin-selected cells transfected with the [0C] sgRNA, as 70.7% of cells contained micronuclei compared with 16.4% of untreated cells (Fig. 4A and B). The frequency decreased as the [C] length increased (Fig. 4A and B), closely paralleling the reduction in ecDNA copy number in Fig. 2. Furthermore, DNA FISH targeting the *MYC* locus revealed that a high proportion of micronuclei contained the *MYC* locus (Fig. 4C and D). In this experiment, cells were analyzed after the puromycin selection, and the proportion of Annexin V-positive apoptotic cells in the micronuclei-positive cells was low irrespective of [C]-extension (Supplementary Fig. S3). These results collectively suggest that cells with high ecDNA copy numbers selectively undergo cell death and that, even in the surviving cells, Cas9-induced DSBs may trigger expulsion of a fraction of ecDNA as micronuclei, potentially contributing to the overall decrease in ecDNA copy number.

While micronuclei were less frequently observed in untreated cells, sgGAL4-transfected cells, and safeguard sgRNA-transfected cells, we found that micronuclei detected in these conditions were also positive for ecDNA signals (Fig. 4C and D). This suggests an inherent uncertainty in ecDNA segregation; however, this effect may be compensated by the increased fitness advantage conferred by ecDNA for cell survival, thereby maintaining high ecDNA copy numbers. Taken together, these results suggest that Cas9-induced DSBs lead to distinct DNA fates: chromosomal DNA is primarily repaired through pathways such as NHEJ, MMEJ, and HR, whereas ecDNA is preferentially lost both through selective cell death of high copy number cells and through micronucleus formation in surviving cells, particularly when targeted with standard sgRNAs (Fig. 4E).

### Computational modeling of cellular consequences of multiple DSBs on multicopy ecDNA

We previously developed computational simulations to understand how safeguard sgRNAs modulate Cas9 activity and HDR efficiency for chromosomal targets, bi-allelic vs mono-allelic editing, and cytotoxicity [26]. To better understand the unique impacts of Cas9-based editing on multicopy ecDNAs, we here developed new computational frameworks that analyze the degree and temporal pattern of multiple DSBs on multicopy ecDNAs. This concept was based on our observation that only a fraction of ecDNA is included in micronuclei (Fig. 4C). For this purpose, we set up new simulations in which Cas9 binding and DSBs are randomly distributed across the defined time frame, and the temporal patterns of Cas9-induced DSB on multicopy ecDNA are recorded for each cell with various ecDNA copy numbers (Fig. 5A). Overview of this computational framework is summarized in Supplementary Fig. S4. In this scheme, [C] extension decreases Cas9 binding and DSB frequency across the time frame in a length-dependent manner (Fig. 5B).

In our simulations, when the sgRNAs can efficiently induce bi-allelic targeting of chromosomal targets, the sgRNAs can target most of ecDNA copies (Fig. 5C and D and Supplementary Fig. S5 and S6). Thus, total amount of DSBs was proportional to ecDNA copy number. In this simulation, standard sgRNAs induced simultaneous DSBs more than five copies in most cells with >50 ecDNA copies (Supplementary Fig. S5C and D). In contrast, safeguard sgRNAs, which induce mono-allelic targeting of chromosomal targets, avoided such temporal convergence of DSB events, and a large fraction of DSBs were temporally isolated (Supplementary Figs S5C, D, and S6).

We next formulated how total amount of DSBs affects the cell death phenotype. Based on previous observations in which the number of target sites (0–20 sites) correlates well with antiproliferative effect of Cas9-mediated DSBs in CRISPR–Cas9 essentiality screens [34–36], we determined a survival function that fitted well with standard sgRNA-induced ecDNA copy number alterations in the CORL23 cell population (Fig. 5E). When the sgRNAs are potent, optimized lethal thresholds are stable: ~33 DSBs are lethal for cells (Fig. 5F, the dashed boxes), thereby preferentially eliminating high copy number cells (Fig. 5G and Supplementary Fig. S7A and B). Under this scheme, the inferred Cas9 binding/DSB frequency linearly correlated with the experimentally validated sgRNA abundance (Fig. 5H and Supplementary Fig. S7C). This was consistent with our previous observations that safeguard sgRNAs suppress Cas9 activity predominantly through inhibition of Cas9 complex formation [26]. The simulated data of ecDNA copy number distribution, cell number, and mean ecDNA copy number adequately fit the experimental results (Fig. 5I–K and Supplementary Fig. S8).

We further computationally analyzed the degree and temporal pattern of multiple DSBs on multicopy ecDNAs in the surviving cells. Under high frequencies of Cas9 binding, while high copy number cells are eliminated, most ecDNA copies are still efficiently targeted in the surviving cells (Supplementary Fig. S9). The presence of unedited sequences in T7E1 and TIDE analyses (Fig. 3) may reflect a subpopulation of cells that failed to express sufficient Cas9. Such effects might be particularly pronounced in the [0C] and [5C] sgRNA conditions, where only a very small fraction of cells survived. This could help explain the discrepancy between our simulation results and the T7E1 assay outcomes shown in Fig. 3B. In addition, ecDNA sequences are often heterogeneous, and we cannot exclude the possibility that ecDNA molecules harboring small modifications at the target site also contribute to the observed unedited signals.

We hypothesized that the pattern of temporal convergence and divergence of multiple DSBs is different between standard and safeguard sgRNAs, leading to frequent micronuclei formation in standard sgRNA experiments. To test this possibility, we examined whether multiple DSBs accumulate within a short time window (Fig. 6A). As expected, we found that higher Cas9 binding frequencies led to a more pronounced accumulation of multiple DSBs (Fig. 6B). Under the [0C] condition, ~6–7 DSBs occurred per five time frames (Fig. 6B). The extent of this accumulation depended on the ecDNA copy number in the surviving cells, consistent with our expectations (Fig. 6C). Therefore, we hypothesized that such clustered multiple DSBs might correlate with micronuclei formation (Fig. 6D). Through parameter optimization (Supplementary Fig. S10), we found that the fraction of cells experiencing 4–5 DSBs per five time frames showed a strong correlation with the proportion of micronuclei-positive cells (Fig. 6E and F).

In addition, based on the report that indel size increases with the overall efficiency of Cas9 mutagenesis [36], we examined whether the frequency of temporally isolated DSBs correlates with the incidence of small indels (Fig. 6G). Interestingly, whereas the total number of DSBs decreased monotonically with increasing [C]-extension, the frequency of temporally isolated DSBs—defined as DSBs not preceded or followed by DSBs on other ecDNA copies—was increased by the safeguard sgRNA (Fig. 6H and I; Supplementary Fig. S11). These simulation results were in good agreement with both the monotonic decrease in indel frequency observed in the T7E1 assay analysis (Fig. 3B) and the rise-and-fall pattern of small indel frequency detected by the TIDE analysis (Fig. 3I). The frequency of temporally isolated DSBs showed a very strong correlation with the TIDE analysis results (Fig. 6I and J). In our simulations, ~44% of temporally isolated DSBs corresponded to the fraction of small indels (Fig. 6J). The generally higher indel frequency detected by T7E1 assay than the small-indel frequency from TIDE analysis also aligns with these obserbations.

Taken together, these simulations suggest that the degree and temporal pattern of multiple DSBs shape the cell death phenotype, micronuclei formation, and partly small indel formation.

### Safeguard sgRNA strategy enables efficient knock-in into multicopy ecDNA while preserving copy number

**Figure 7.**
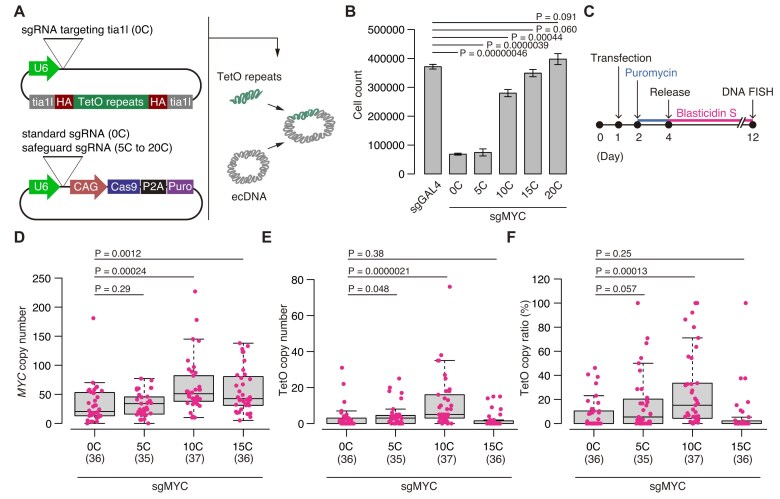
ecDNA knock-in efficiency of standard and safeguard sgRNAs. (**A**) Schematic showing insertion of a 96-mer TetO repeat sequence into ecDNA of CORL23 cells. See Material and Methods for details of the knock-in system. (**B**) Quantification of CORL23 cell numbers after expression of all-in-one CRISPR plasmids with the indicated sgRNAs and the knock-in template plasmid. Data represent mean ± SD for *n* = 3 biological replicates. Statistical significance was assessed using a two-sided Student’s *t*-test. (**C**) Experimental scheme for DNA FISH analyses shown in panels (D–F). (**D**–**F**) Box-and-whisker plots showing copy numbers of (**D**) *MYC* ecDNA and (**E**) TetO knock-in ecDNA in cells transfected with all-in-one CRISPR plasmids with the indicated sgRNAs and the knock-in template plasmid. Panel (**F**) shows the fraction of *MYC* ecDNA carrying the TetO knock-in sequence. Sample sizes (*n*) are shown below sgRNA labels. Statistical significance was assessed using a Wilcoxon rank-sum test.

In our previous study, the safeguard sgRNA approach improved the efficiency of scarless monoallelic cassette knock-in in mESCs and the efficiency of generating and correcting disease-associated single-nucleotide substitutions in mESCs and human iPSCs using single-stranded oligodeoxynucleotide HDR templates [26]. We therefore investigated the performance of the standard and safeguard sgRNA approaches in knock-in experiments using HDR. Following previous reports [7, 15], we designed knock-in experiments in which a 96-mer TetO array was inserted into *MYC* ecDNA of CORL23 cells for visualization with fluorescently tagged TetR proteins (Fig. 7A). Similar to the simple Cas9 expression experiments (Fig. 2), expression of all-in-one CRISPR plasmids with the [0C] sgRNA together with HDR template plasmids resulted in severe growth impairment (Fig. 7B). A similar reduction was also observed with the [5C] sgRNA. By contrast, [10C–20C] safeguard sgRNAs improved viability (Fig. 7B). We next measured the numbers of *MYC* ecDNA and TetO-knock-in *MYC* ecDNA using FISH probes for the *MYC* locus and TetO sequence (Fig. 7C–F and Supplementary Fig. S12). Because the TetO cassette carried a blasticidin S resistance gene, cells were cultured with blasticidin S after puromycin selection (Fig. 7C). Consistent with the cytotoxicity results (Fig. 7B), cells edited with the [0C] sgRNA exhibited low ecDNA copy numbers, and a similar decrease was also observed with the [5C] sgRNA (Fig. 7D). In contrast, copy number began to recover with the [10C] sgRNA (Fig. 7D). The same trend was observed when cells were cultured without blasticidin S (Supplementary Fig. S12A and B). Together, these results suggest that, for HDR template–mediated editing of *MYC* ecDNA in CORL23 cells, safeguard sgRNAs are preferable because they reduce cytotoxicity and prevent ecDNA loss.

Next, we analyzed both the absolute number and relative fraction of *MYC* ecDNA carrying TetO repeats in individual cells using two-color FISH, in which *MYC* and TetO loci were simultaneously detected (Fig. 7E and F). Counting TetO-positive ecDNA copies showed that both the number and the fraction were low with [0C] and [15C] sgRNAs (Fig. 7E and F). Although slightly increased with the [5C] sgRNA, the number and fraction still remained low. By contrast, cells expressing the [10C] sgRNA contained more TetO-positive ecDNAs per cell than under other conditions, demonstrating superior knock-in efficiency (Fig. 7E and F). In this setting, [10C] was the most favorable condition for knock-in efficiency. When cultured without blasticidin S, the fraction of knock-in ecDNA was very low (median < 1%) in most cells across all conditions (Supplementary Fig. S12C and D), suggesting that selection with the knock-in cassette is necessary for enrichment of knock-in cells.

All knock-in experiments were performed using all-in-one plasmids with the CAG promoter.

**Figure 8.**
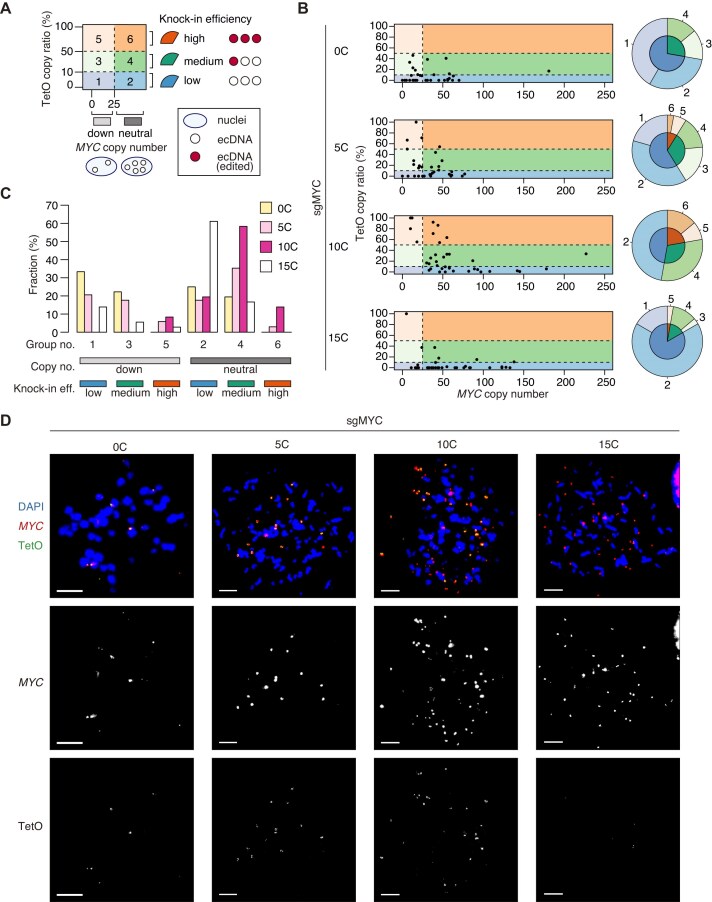
Relationship between ecDNA copy number and TetO knock-in efficiency. (**A**) Schematic showing the classification of individual cells into six groups according to *MYC* ecDNA copy number and the fraction of TetO-positive ecDNA. (**B**) Scatter plots of individual cells grouped as in panel (A), with accompanying pie charts showing the proportion of cells in each group. (**C**) Summary of group distributions. (**D**) Representative DNA FISH images. Scale bar, 10 µm.

To examine the relationship between overall *MYC* ecDNA copy number (Fig. 7D) and the fraction of TetO-positive ecDNA (Fig. 7F), we classified individual cells into six groups as outlined in Fig. 8A. Groups 1 and 2 correspond to cells with low knock-in efficiency (<10%); Groups 3 and 4 represent intermediate efficiency (>10% and <50%); and Groups 5 and 6 represent high efficiency (>50%), as defined by the fraction of TetO-positive ecDNA (y-axis in Fig. 8A). In addition, Groups 1, 3, and 5 correspond to cells with low ecDNA copies (<25 copies), whereas Groups 2, 4, and 6 preserved high copy numbers (>25 copies) (*x*-axis in Fig. 8A). This classification aids in the interpretation of knock-in outcomes. For example, Group 1, with both low ecDNA copies and low knock-in efficiency, represents an unfavorable outcome, and a high proportion of cells in this group suggests poor editing performance. By contrast, Group 6, with high copies and high knock-in efficiency, represents the most desirable outcome. Group 4, although showing only intermediate knock-in efficiency, maintains high ecDNA copies and is therefore relatively favorable. Thus, conditions in which cells are enriched in Groups 4 and 6 can be considered successful conditions for ecDNA knock-in.

As quantified in Fig. 8B and C (see also Supplementary Fig. S13), cells transfected with [0C] sgRNA were mainly classified into Groups 1 and 2, indicating marked loss of ecDNA and low knock-in efficiency. Notably, Group 6 cells were not obtained with [0C] sgRNA. With [5C] sgRNA, the distribution was similar, but a small fraction of cells appeared in Groups 5 and 6. In [10C] sgRNA conditions, the most unfavorable Group 1 was absent, whereas Group 2 increased. Importantly, Groups 4 and 6 became prominent with [10C] sgRNA, representing efficient knock-in and preservation of ecDNA copy number. By contrast, [15C] sgRNA resulted in an increased proportion of Group 2 but a decline in Groups 4 and 6, indicating reduced knock-in efficiency, consistent with reduced DSB induction. These results indicate that, within our experimental framework, [10C] sgRNA provided the most favorable outcome for ecDNA knock-in. Representative two-color FISH images are shown in Fig. 8D, demonstrating that a subset of safeguard sgRNAs enable highly efficient knock-in of TetO sequences into ecDNA while preserving copy number—an outcome rarely achieved with conventional sgRNAs.

### Computational inference of rapid expansion of knock-in ecDNA with safeguard sgRNAs

**Figure 9.**
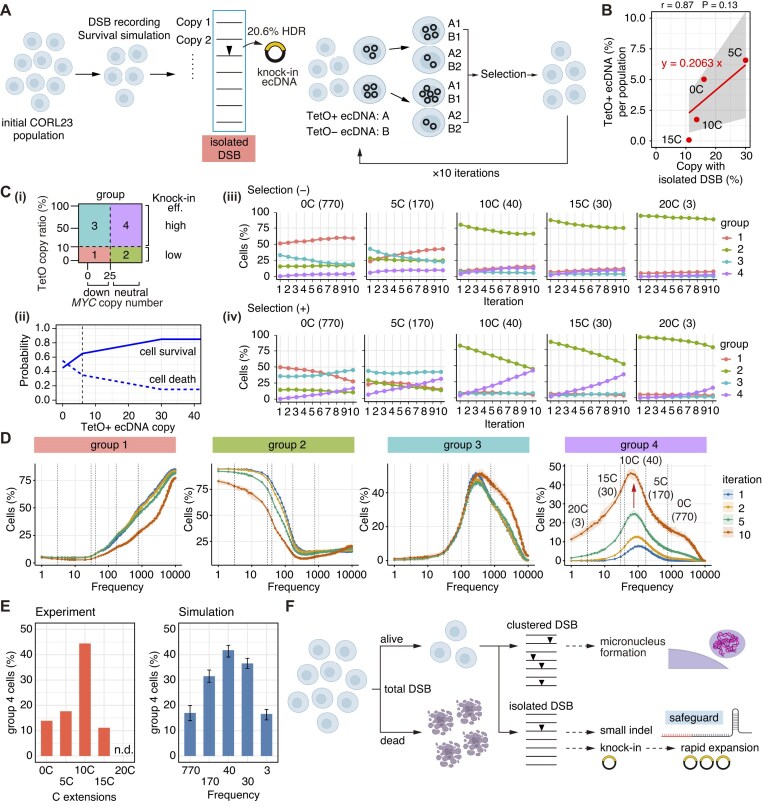
Computational inference of rapid expansion of knock-in ecDNA. (**A**) Scheme for simulations of uneven segregation of ecDNA and blasticidin S selection. (**B**) Correlation between the proportion of knock-in ecDNA under blasticidin S (−) condition (Supplementary Fig. S12) and the simulated copy fraction with temporally isolated single DSBs. Linear regression curves, Pearson’s correlation coefficients (*r*) with 95% confidence intervals, and *P* values are shown. (**C**) Simulations of cell dynamics. (i) Classification of individual cells into four groups according to *MYC* ecDNA copy number and the fraction of TetO-positive ecDNA. (ii) Scheme of blasticidin S selection. (iii) Cell dynamics under neutral selection. (iv) Cell dynamics under blasticidin S selection shown in (ii). (**D**) Simulations of cell dynamics under various frequencies of Cas9 binding. The shaded area represents the max–min range. The dotted lines represent the inferred frequencies of Cas9 binding for each sgRNA. (**E**) Comparison of the fraction of high ecDNA copy and high-level knock-in cells in experiments (left) and simulations (right). The error bar represents the max–min range. n.d., not determined. (**F**) Summary of computational simulations.

By expanding the computational framework in Figs 5 and 6, we next examined how the safeguard sgRNAs enable the emergence of high ecDNA copy and high-level knock-in cells. We tested the effects of uneven segregation of ecDNA on expansion of knock-in ecDNA by combining the previous simulations and additional simulation of ecDNA segregation (Fig. 9A).

While the fraction of knock-in ecDNA was very low in the absence of blasticidin S (Supplementary Fig. S12), the knock-in fraction among total ecDNA copies correlated well with the fraction of copies with temporally isolated DSBs (Fig. 9B), as temporally isolated DSBs correlated well with the fraction of small indels (Fig. 6I and J). In our simulations, ~21% of temporally isolated DSBs corresponded to the fraction of knock-in ecDNA in the absence of blasticidin S (Fig. 9B). This frequency was consistent with the reported HDR rate [42]. Thus, we reconstructed the surviving CORL23 population by converting ecDNA with temporally isolated DSBs to knock-in ecDNA at a rate of 21% and then subjected these cells to the cycle of uneven segregation of ecDNA and blasticidin S selection (Fig. 9A), and monitored ecDNA copy number dynamics. As shown in Fig. 9C, copy number status did not largely change under neutral selection, but high ecDNA copy and high-level knock-in cells rapidly expanded under blasticidin S selection when the Cas9 binding frequency was adjusted to [10C] sgRNA conditions. Based on the copy number distribution in the absence or presence of blasticidin S, we defined the selective advantage (Supplementary Fig. S14). Importantly, the same trend was reproduced with different schemes of selective advantage (Supplementary Fig. S15). By carefully monitoring the cell status under various Cas9 binding frequencies, we conspicuously observed that safeguard sgRNAs enable rapid expansion of high ecDNA copy and high-level knock-in cells (Fig. 9D). This was attributable to generation of rare high ecDNA copy and high-level knock-in cells with safeguard sgRNAs (Fig. 9D, group 4, iteration 1). The fraction of high ecDNA copy and high-level knock-in cells is comparable between experiments and simulations (Fig. 9E). Taken together, these simulations provide mechanistic insights into rapid expansion of knock-in ecDNA with safeguard sgRNA (Fig. 9F).

### Stability and visualization of TetO knock-in ecDNA

**Figure 10.**
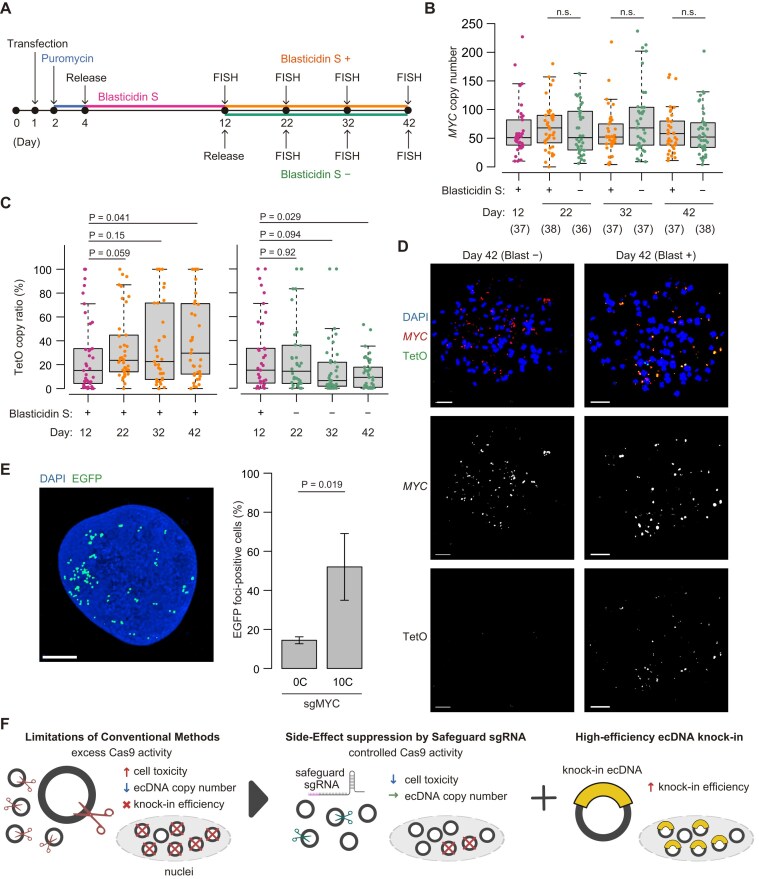
Stability and visualization of TetO knock-in ecDNA. (**A**) Schematic of the experimental timeline. (**B**) Box-and-whisker plots showing copy numbers of total *MYC* ecDNA in cells cultured with or without blasticidin S. Statistical significance was assessed using a Wilcoxon rank-sum test. (**C**) Box-and-whisker plots showing fractions of the TetO knock-in ecDNA in cells cultured with (left) or without (right) blasticidin S. Statistical significance was assessed using a Wilcoxon rank-sum test. (**D**) Representative DNA FISH images. Scale bar, 10 µm. (**E**) Visualization of TetO knock-in ecDNA using TetR-EGFP. (**F**) Graphical summary of the present study.

Finally, we assessed the stability of TetO knock-in ecDNA obtained with the [10C] sgRNA condition, which exhibited the highest knock-in efficiency. Cells were cultured either with or without blasticidin S, and the copy number of knock-in ecDNA was measured at multiple time points (Fig. 10A). First, regardless of blasticidin S treatment, the copy number of *MYC* ecDNA remained unchanged over time (Fig. 10B). However, when cultured without blasticidin S, TetO knock-in ecDNA showed a tendency to decrease over time, whereas continuous blasticidin S treatment led to an increase (Fig. 10C and D). These findings suggest the dynamic and plastic nature of ecDNA sequences, while their persistence is promoted by the increased fitness as they confer for cell survival. Therefore, sustained selection is required to maintain the copy number of TetO-positive ecDNA. Finally, when TetR-EGFP was expressed in a bulk population of cells obtained from [10C] sgRNA knock-in experiments and maintained under blasticidin S selection, ecDNA became visible in a substantial fraction of cells, validating the effectiveness of our experimental strategy (Fig. 10E).

## Discussion

In this study, we systematically compared the performance of the CRISPR-Cas9 system with standard sgRNAs and safeguard sgRNAs for the engineering of multicopy ecDNA in cancer cells. Although a few studies have reported knock-in approaches targeting ecDNA [7, 15], these studies did not address the efficiency and potential pitfalls. Use of conventional sgRNAs induced pronounced cytotoxicity and copy number loss in ecDNA-positive cells, resulting in low indel and knock-in efficiency (Fig. 10F), consistent with previous reports [7, 18, 43]. Under these conditions, phenotypic changes caused by ecDNA copy number loss should be carefully assessed. By contrast, safeguard sgRNAs suppressed these adverse effects and enabled controlled knock-in of multicopy ecDNA (Fig. 10F).

In our previous study, severe cytotoxicity was induced by standard sgRNAs in human iPSCs, which are highly sensitive to DSB-mediated p53 activation. Such cytotoxicity in iPSCs was induced by targeting various chromosomal sequences and was alleviated by safeguard sgRNAs, which enabled correction of disease-associated single-nucleotide substitutions via HDR [26]. CORL23 cells are tolerant of chromosomal targeting (Fig. 2, see Chr2 targeting), and this can be partly explained by p53 mutation in CORL23 cells [44, 45]. Consistent with the previous reports showing that the number of target sites (0–20 copies) correlates well with the antiproliferative effect of Cas9-mediated DSBs in CRISPR–Cas9 essentiality screens [34–36], our simulations confirmed that multiple DSBs of ecDNA account for ecDNA editing–induced cytotoxicity and elimination of high copy number cells. Although the effect may be modest, micronuclei formation might also contribute to the reduction in ecDNA copy number. These findings are consistent with an earlier report in COLO320DM cells [18], showing that damaged ecDNA aggregates and is expelled from the nucleus via micronucleus formation [18]. A similar phenomenon was reported after hydroxyurea (HU) treatment in ecDNA-positive cells [46]. Our simulation analyses indicate that efficient genome editing of ecDNA is more likely to induce cell death and/or micronuclei formation as the number of target copies increases. This suggests that ecDNA loss is not a cell line–specific phenomenon but a general outcome of conventional genome editing of ecDNA, underscoring its fundamental limitations.

In this study, the safeguard sgRNA strategy markedly improved cell viability and preserved ecDNA copy number. Importantly, we found that the length of the [C] extension strongly influenced the outcome. [0C] or [5C] sgRNAs caused severe cytotoxicity, ecDNA loss, and poor knock-in efficiency. By contrast, a longer extension such as [15C] sgRNA suppressed toxicity but appeared to excessively inhibit Cas9 activity, resulting in inefficient editing. Strikingly, the [10C] sgRNA balanced these effects, allowing efficient knock-in while maintaining ecDNA copy number.

The improved knock-in efficiency observed with [10C] sgRNA may be interpreted as a consequence of more regulated and less rampant DSB induction. A previous study showed that inducible expression of Cas9 rapidly triggered ecDNA aggregation within a few hours [18], highlighting the sensitivity of ecDNA to acute DSB formation across multiple copies.

In our computational modeling, we demonstrated that standard sgRNAs and safeguard sgRNAs generate two opposing temporal patterns: temporally clustered DSBs and temporally isolated DSBs, respectively (Fig. 9F). Moreover, the frequency of temporally clustered DSBs was associated with micronuclei formation, whereas the frequency of temporally isolated DSBs was linked to small indel formation and the rapid expansion of knock-in ecDNA after selection. Mechanistically, temporally clustered DSB events are expected to promote aggregation of damaged ecDNA fragments and subsequent micronucleus expulsion. Notably, condensation phenomena at sites of DNA damage have also been observed for chromosomal DNA, where repair factors assemble into local condensates to tether broken ends together and facilitate repair [47]. In the context of ecDNA, extensive and synchronous DSB-induced condensates may drive large-scale clustering and subsequent cell death or loss of DNA molecules. By contrast, safeguard sgRNAs attenuate the formation of functional Cas9 complexes, thereby reducing the probability of temporally clustered DSBs across numerous ecDNA copies and increasing the probability of temporally isolated DSBs. This adjustment may prevent catastrophic aggregation and instead permit individual breaks to undergo small indel formation or HDR, depending on the presence of an HDR template. How the balance between temporally clustered DSBs and temporally isolated DSBs influences the balance of post-cleavage outcomes—such as small indel formation versus larger indel formation or complex rearrangements—will need to be examined in future studies. In addition, our study revealed that the characteristic uneven segregation of ecDNA enables the rapid expansion of knock-in ecDNA. We further showed that this expansion is enhanced by safeguard sgRNAs, and these findings mechanistically reinforce the usefulness of safeguard sgRNAs in ecDNA knock-in.

There are several limitations in our analysis. Achieving knock-in across all copies of ecDNA remains difficult, given the large and heterogeneous population of ecDNA molecules within a single cell. Moreover, because of this high copy numbers, it is technically challenging to verify the precise sequence status of individual ecDNA molecules after knock-in using conventional sequencing approaches. In addition, although [10C] sgRNA emerged as optimal in our experimental setting, the most effective length of [C] extension may vary depending on the target sequence, knock-in template, and cellular context. Nonetheless, the fundamental advantage of employing safeguard sgRNAs is likely to be broadly applicable across diverse ecDNA-positive cells.

In conclusion, we provide evidence that ecDNA is highly sensitive to perturbation by conventional genome editing and show that safeguard sgRNAs constitute a useful strategy for engineering ecDNA in various molecular biology applications, thereby advancing our understanding of ecDNA biology. The present study also demonstrates that continuous modulation of Cas9 activity with safeguard sgRNAs is important to study the DSB response of ecDNA and its cellular consequences.

## Supplementary Material

gkag005_Supplemental_Files

## Data Availability

All data supporting the findings of this study are available within the article and its supplementary information or will be made available from the authors upon request.
