# Correction to ‘Optimized CRISPR-Cas9 system for efficient engineering of ecDNA in cancer cells’

**DOI:** 10.1093/nar/gkag498

**Published:** 2026-05-15

This is a correction to: Yohei Sugimoto, Takeru Kachi, Yu Watanabe, Mei Kubokawa, Koichi Ogami, Masaki Kawamata, Seiko Yoshino, Hiroshi I Suzuki, Optimized CRISPR-Cas9 system for efficient engineering of ecDNA in cancer cells, *Nucleic Acids Research*, Volume 54, Issue 2, 27 January 2026, gkag005, https://doi.org/10.1093/nar/gkag005

In the originally published version of the paper there was an error in the assignment of panel letters in the legend of Figure 5. The description of panel C is errored and the letters used from (C) to (H). Text in the legend is corrected in the article to read: “(**D**) Cell-level quantification of total amount of DSBs (left) and max DSB number per one time frame (right) across various frequencies of Cas9 binding and DSB events. The dotted lines represent the inferred frequencies of Cas9 binding for each sgRNA, and the corresponding sgRNAs are indicated in panel (G). (**E**) Scheme for total DSB-dependent cytotoxicity. (**F**) Identification of the lethal thresholds that minimized SSR for the results of standard sgRNA ([0C] sgRNA). Left panel shows the relationships among lethal threshold, SSR, and frequencies of Cas9 binding and DSB events. Note that lethal threshold is constant for a wide range of highly efficient sgRNA (lethal threshold = 33, see the dashed boxes). (**G**) Changes in cell fraction in the CORL23 cell population under various frequencies of Cas9 binding. (**H**) Correlation between sgRNA abundance and inferred frequencies of Cas9 binding for each sgRNA. The dotted lines represent the inferred frequencies of Cas9 binding for each sgRNA. (**I**) Comparison of ecDNA copy number distribution in experiments (top) and simulations (bottom).” instead of: “Cell-level quantification of total amount of DSBs (left) and max DSB number per one time frame (right) across various frequencies of Cas9 binding and DSB events. The dotted lines represent the inferred frequencies of Cas9 binding for each sgRNA, and the corresponding sgRNAs are indicated in panel (G). (**D**) Scheme for total DSB-dependent cytotoxicity. (**E**) Identification of the lethal thresholds that minimized SSR for the results of standard sgRNA ([0C] sgRNA). Left panel shows the relationships among lethal threshold, SSR, and frequencies of Cas9 binding and DSB events. Note that lethal threshold is constant for a wide range of highly efficient sgRNA (lethal threshold = 33, see the dashed boxes). (**F**) Changes in cell fraction in the CORL23 cell population under various frequencies of Cas9 binding. (**G**) Correlation between sgRNA abundance and inferred frequencies of Cas9 binding for each sgRNA. The dotted lines represent the inferred frequencies of Cas9 binding for each sgRNA. (**H**) Comparison of ecDNA copy number distribution in experiments (top) and simulations (bottom).” The description of the legend of Figures 3, 5, and 7 is also updated.

