# Supplementary material for: Optimized CRISPR-Cas9 system for efficient engineering of ecDNA in cancer cells
Source: Nucleic Acids Res. 2026 Jan 14;54(2):gkag005. doi: 10.1093/nar/gkag005 (PMC12802944; doi:10.1093/nar/gkag005)
Supplement: gkag005_Supplemental_Files [file gkag005_supplemental_files.zip › Supplementary_Figure_submit.pdf]

SUPPLEMENTARY INFORMATION

Optimized CRISPR-Cas9 system for efficient engineering of ecDNA in cancer cells

Yohei Sugimoto, Takeru Kachi, Yu Watanabe, Mei Kubokawa, Koichi Ogami, Masaki Kawamata, Seiko Yoshino, Hiroshi I. Suzuki

Supplementary Table

Supplementary Table S1: Oligonucleotide sequences.

Supplementary Figures

Supplementary Figure S1: Validation of the all-in-one ecTag vector for ecDNA visualization.
Supplementary Figure S2: Design of all-in-one CRISPR plasmids expressing standard and safeguard sgRNA and qPCR primers.
Supplementary Figure S3: Relationship between micronucleus formation and apoptosis.
Supplementary Figure S4: Overview of computational framework in this study.
Supplementary Figure S5: Cell-level quantification of multiple DSBs on multicopy ecDNA.
Supplementary Figure S6: Copy-level quantification of multiple DSBs on multicopy ecDNA.
Supplementary Figure S7: Simulation of cell death and Cas9-binding frequencies.
Supplementary Figure S8: Simulation of ecDNA copy number distribution.
Supplementary Figure S9: Simulation results of Cas9-induced in the surviving
Supplementary Figure S10: Computational simulations of clustered multiple DSBs.
Supplementary Figure S11: Dynamics of single vs multiple DSBs.
Supplementary Figure S12: Analysis of ecDNA copy number and knock-in efficiency without blasticidin S selection.
Supplementary Figure S13: Relationship between ecDNA copy number and TetO knock-in copy number.
Supplementary Figure S14: Comparison of TetO knock-in ecDNA copy distribution in the absence and presence of blasticidin S.
Supplementary Figure S15: Simulations of cell dynamics with different selection schemes.

Supplementary Figure S1

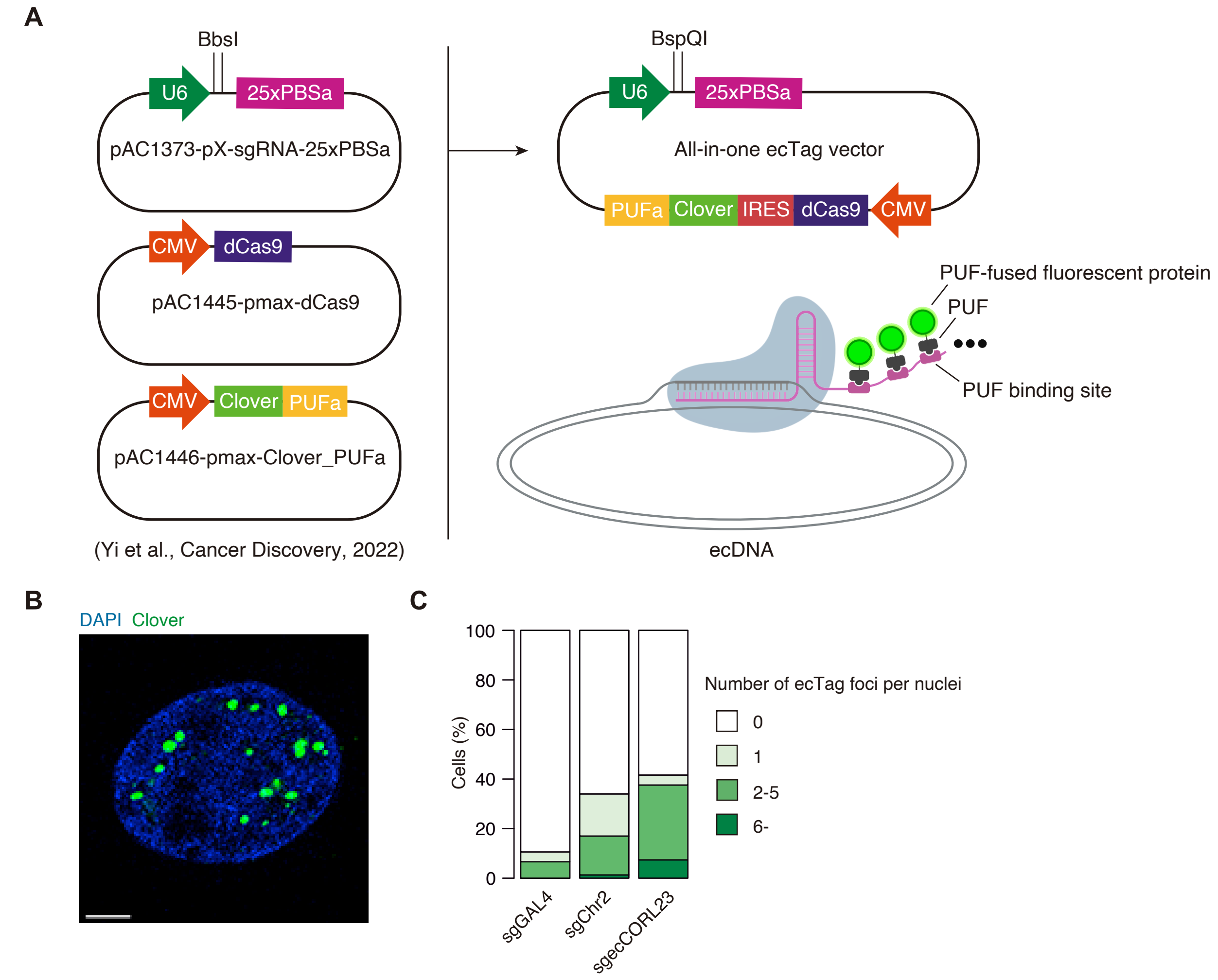

Supplementary Figure S1: Validation of the all-in-one ecTag vector for ecDNA visualization.

(A) Schematic overview of the construction of the all-in-one ecTag vector.

(B) Representative image of CORL23 cells transfected with the all-in-one ecTag vector.

(C) Quantification of Clover signal foci per cell in CORL23 cells expressing the all-in-one ecTag vector with the indicated sgRNAs. Sample sizes (n) were 151 (sgGAL4), 153 (sgChr2), and 149 (sgecCORL23).

Created in BioRender. Sugimoto, Y. (https://BioRender.com/80jzgbz).

Supplementary Figure S2

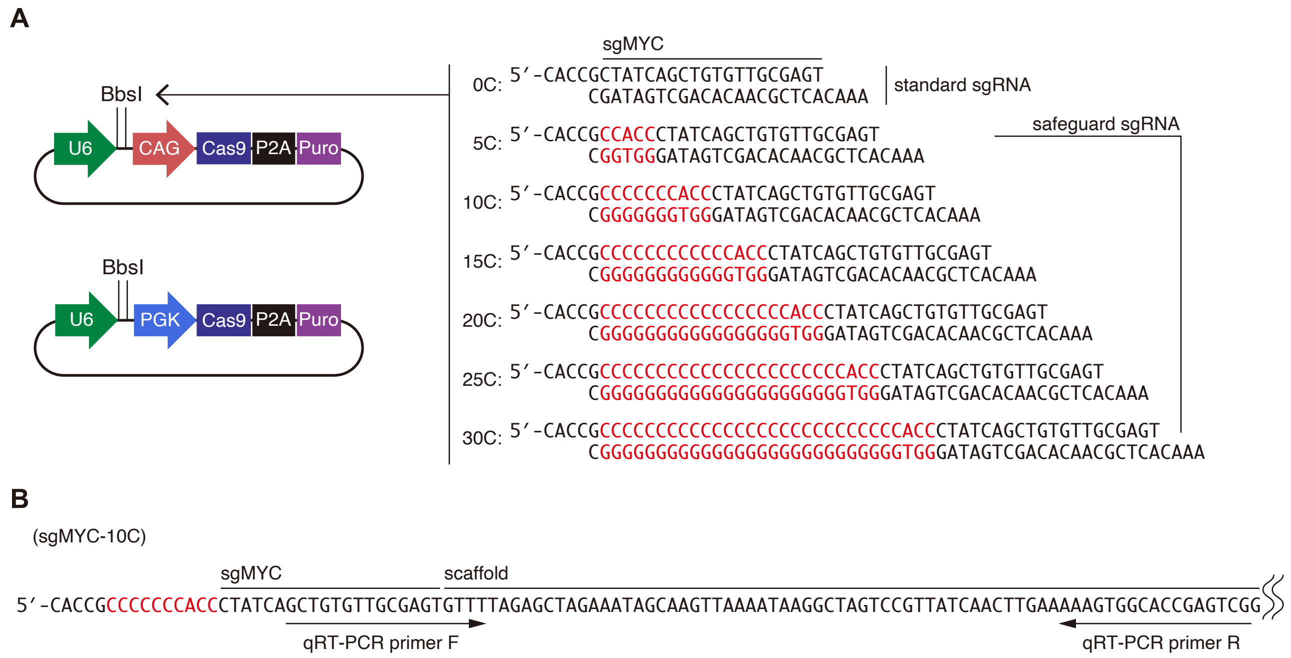

Supplementary Figure S2: Design of all-in-one CRISPR plasmids expressing standard and safeguard sgRNAs and qPCR primers.

(A) Schematic representation of the all-in-one CRISPR plasmids expressing standard and safeguard sgRNAs.

(B) qPCR primer design for quantifying sgRNA expression.

Supplementary Figure S3

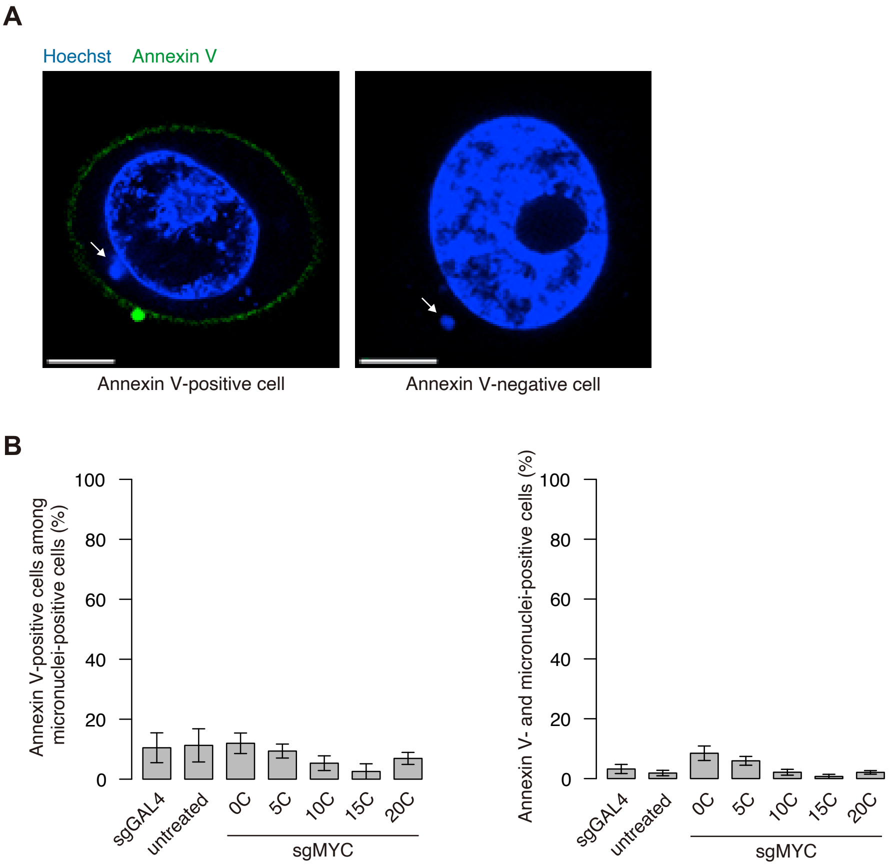

Supplementary Figure S3: Relationship between micronucleus formation and apoptosis.

(A) Representative images of micronuclei-positive CORL23 cells stained with Annexin V. White arrows indicate micronuclei. Scale bar, 10 μm.

(B) Left, Proportion of Annexin V-positive cells among micronuclei-positive CORL23 cells (left). Data represent mean ± SD for n = 3 biological replicates. The numbers of cells analyzed were 116 (sgGAL4), 124 (untreated), 116 ([0C]), 128 ([5C]), 129 ([10C]), 116 ([15C]), and 129 ([20C]). Right, Approximate proportion of both micronuclei- and Annexin V-positive cells among total CORL23 cells, calculated based on the results shown in Fig. 4B and panel (B, left). Data represent mean ± SD. All experiments in this figure were performed using all-in-one vector with CAG promoter.

Supplementary Figure S4

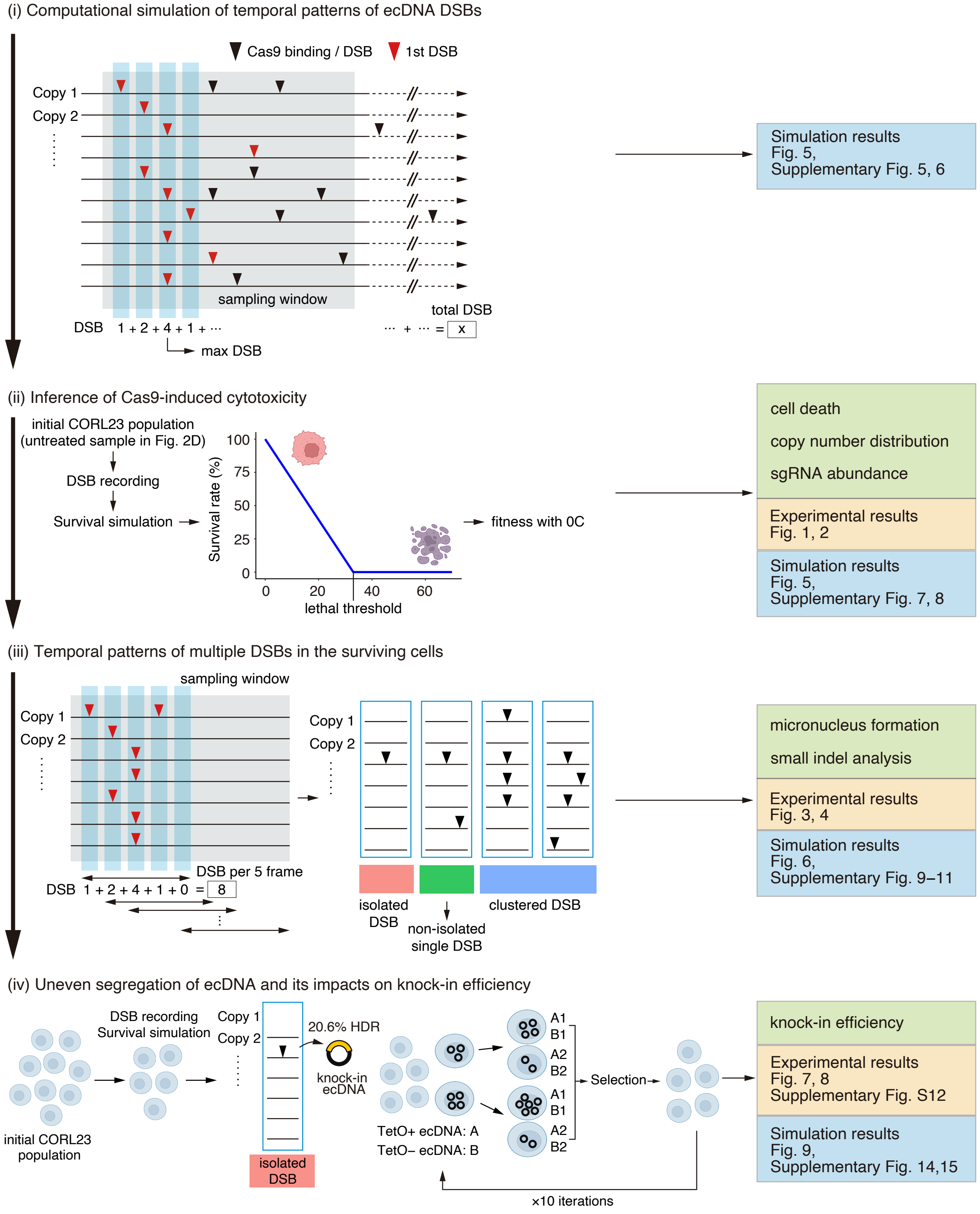

Supplementary Figure. S4: Overview of computational framework in this study. The details are described in the Material and Methods section.

Created in BioRender. Sugimoto, Y. (https://BioRender.com/mpmsanh and https://BioRender.com/sm21gi2).

Supplementary Figure S5

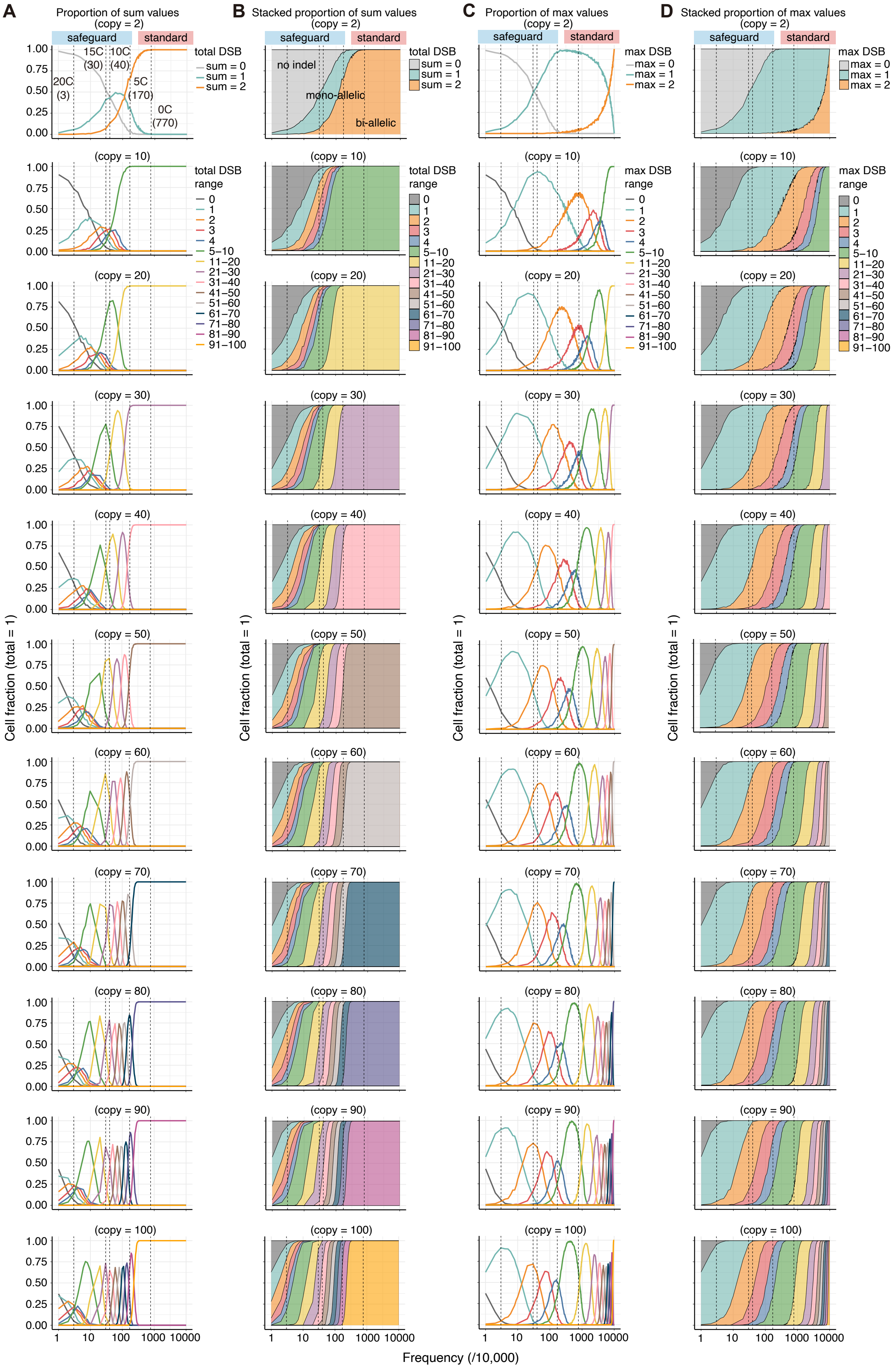

Supplementary Figure. S5: Cell-level quantification of multiple DSBs on multicopy ecDNA.

(A, B) Non-stacked (A) and stacked (B) plots showing the cell fractions stratified by total DSBs under various Cas9 binding frequencies.

(C, D) Non-stacked (C) and stacked (D) plots showing the cell fractions stratified by max DSB number per one time frame across the entire sampling window under various Cas9 binding frequencies.

The plots for copy number 2, 10, 50, and 100 are displayed in Fig. 5D. The dotted lines represent the inferred frequencies of Cas9 binding for each sgRNA, and the corresponding sgRNAs are indicated in the top of (A).

Supplementary Figure S6

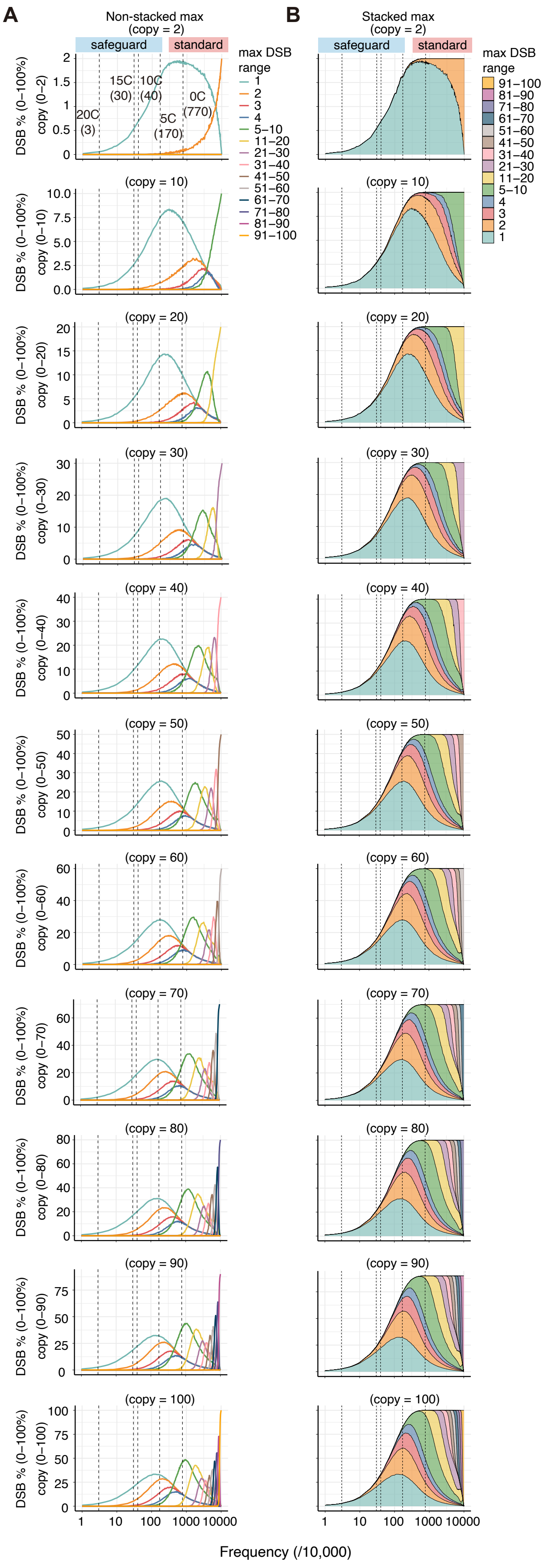

Supplementary Figure. S6: Copy-level quantification of multiple DSBs on multicopy ecDNA.

(A, B) Non-stacked (A) and stacked (B) plots showing the breakdown of the maximum DSB number in copies that experienced DSBs under various Cas9 binding frequencies.

The dotted lines represent the inferred frequencies of Cas9 binding for each sgRNA, and the corresponding sgRNAs are indicated in the top of (A).

Supplementary Figure S7

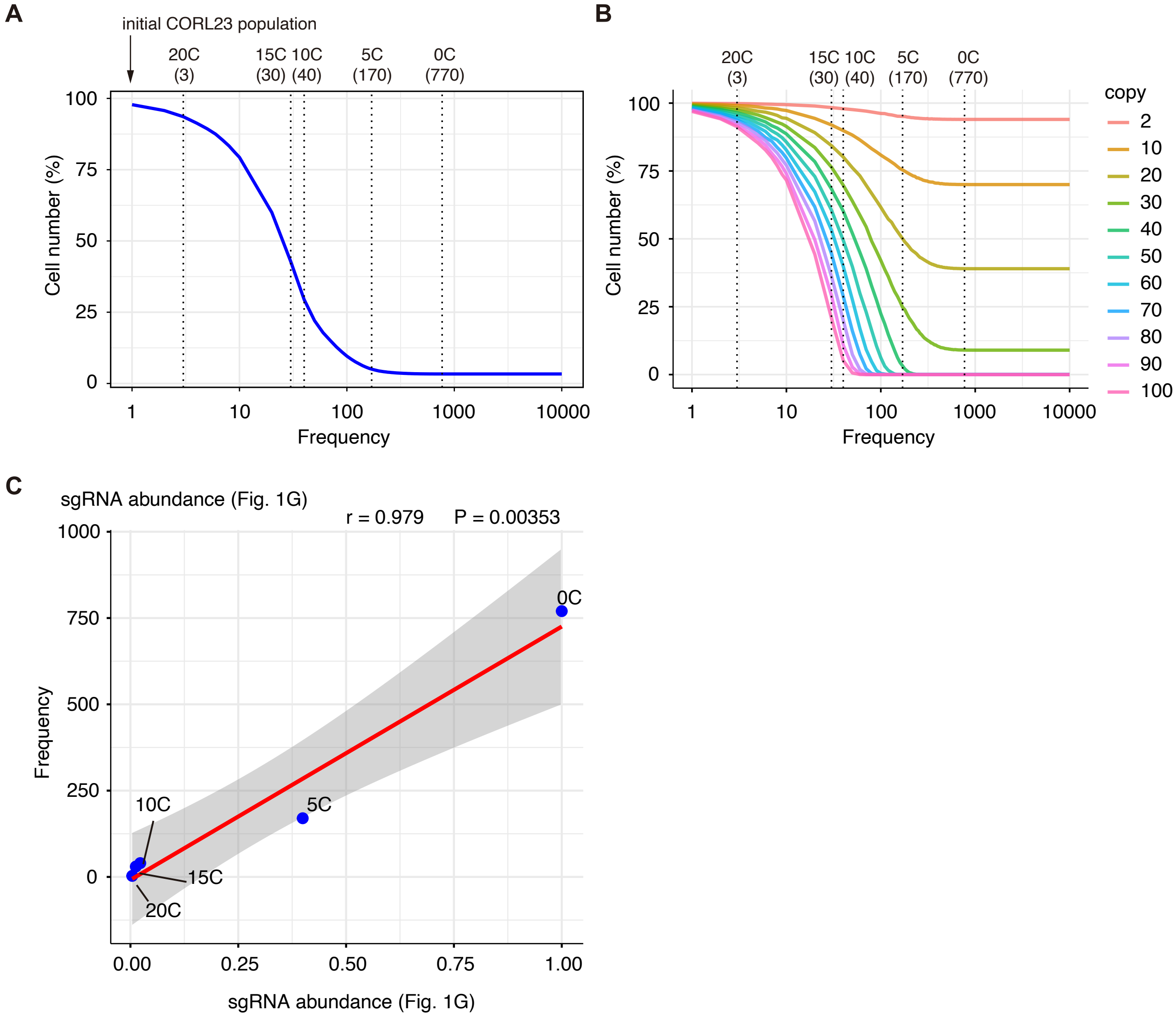

Supplementary Figure. S7: Simulation of cell death and Cas9-binding frequencies.

(A) Simulation of cell number of CORL23 cells under various Cas9 binding frequencies.

(B) Simulations of cell number of cells with various ecDNA copy numbers under various Cas9 binding frequencies.

(C) Correlation between sgRNA abundance (Fig. 1G) and inferred frequencies of Cas9 binding for each sgRNA. Log-scale display is shown in Fig. 5H. Linear regression curves, Pearson's correlation coefficients (r) with 95% confidence intervals and P values are shown.

In (A and B), the dotted lines represent the inferred frequencies of Cas9 binding for each sgRNA.

Supplementary Figure S8

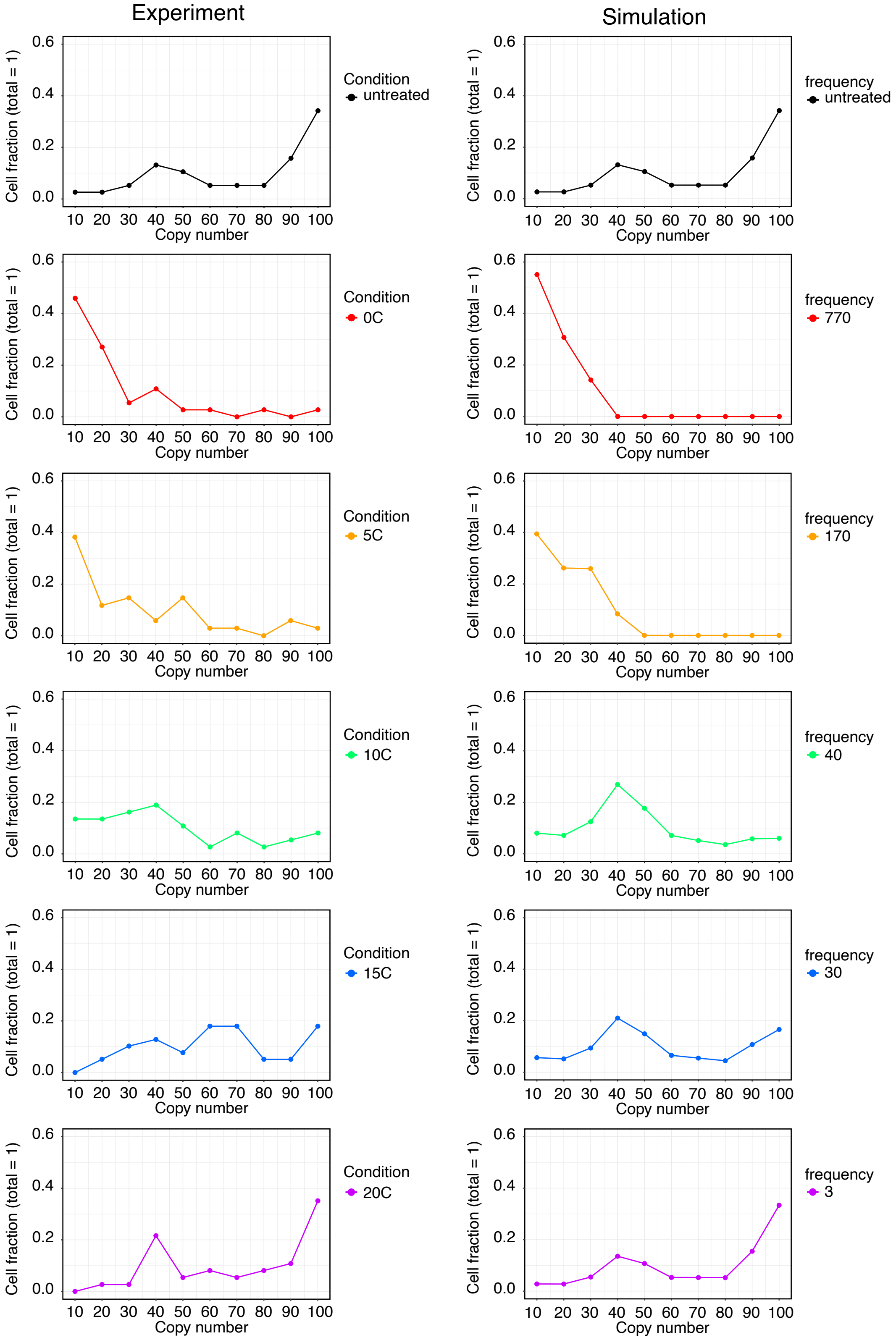

Supplementary Figure. S8: Simulation of ecDNA copy number distribution.

Comparison of ecDNA copy number distribution in experiments (left) and simulations (right) is shown.

Supplementary Figure S9

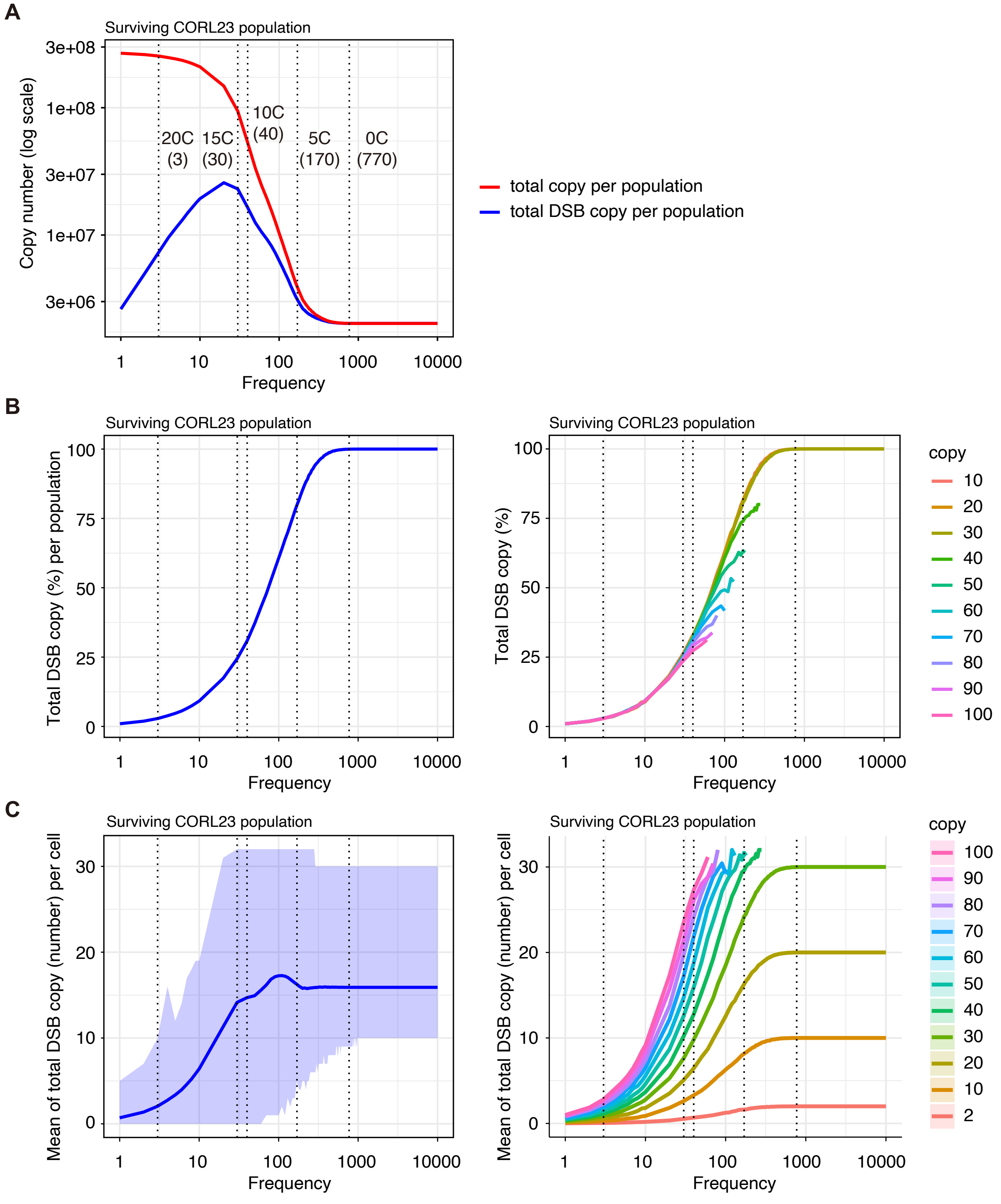

Supplementary Figure. S9: Simulation results of Cas9-induced DSBs in the surviving CORL23 population.

(A) Changes in total ecDNA number and DSB-positive ecDNA number under various Cas9 binding frequencies.

(B) Proportion of DSB-positive ecDNA copies under varying Cas9 binding frequencies (left). Right panel displays separately the results for cells grouped by ecDNA copy number.

(C) Number of DSB-positive ecDNA copies under varying Cas9 binding frequencies (left). The shaded area represents the max–min range. Right panel displays separately the results for cells grouped by ecDNA copy number.

In (A–C), the dotted lines represent the inferred frequencies of Cas9 binding for each sgRNA, and the corresponding sgRNAs are indicated in (A).

Supplementary Figure S10

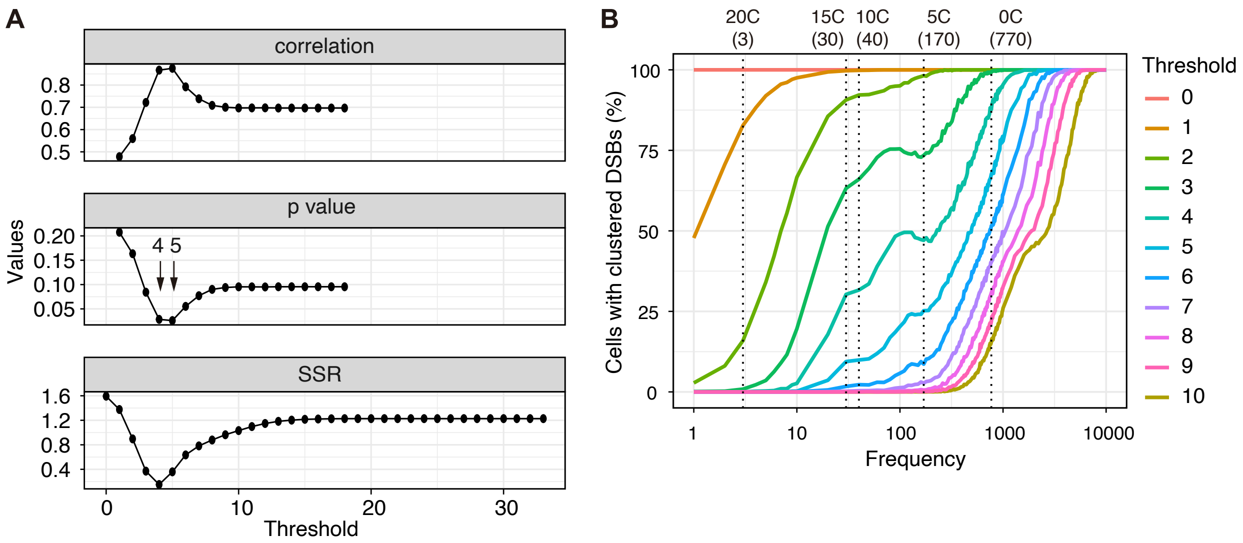

Supplementary Figure. S10: Computational simulations of clustered multiple DSBs.

(A) Computational estimation of micronuclei-positive cells. In the micronuclei analysis, we calculated the correlation and SSR between the fraction of micronuclei-positive cells and the fraction of cells exhibiting clustered multiple DSBs using different thresholds.

(B) Proportion of cells with clustered multiple DSBs under different thresholds. The dotted lines represent the inferred frequencies of Cas9 binding for each sgRNA.

Supplementary Figure S11

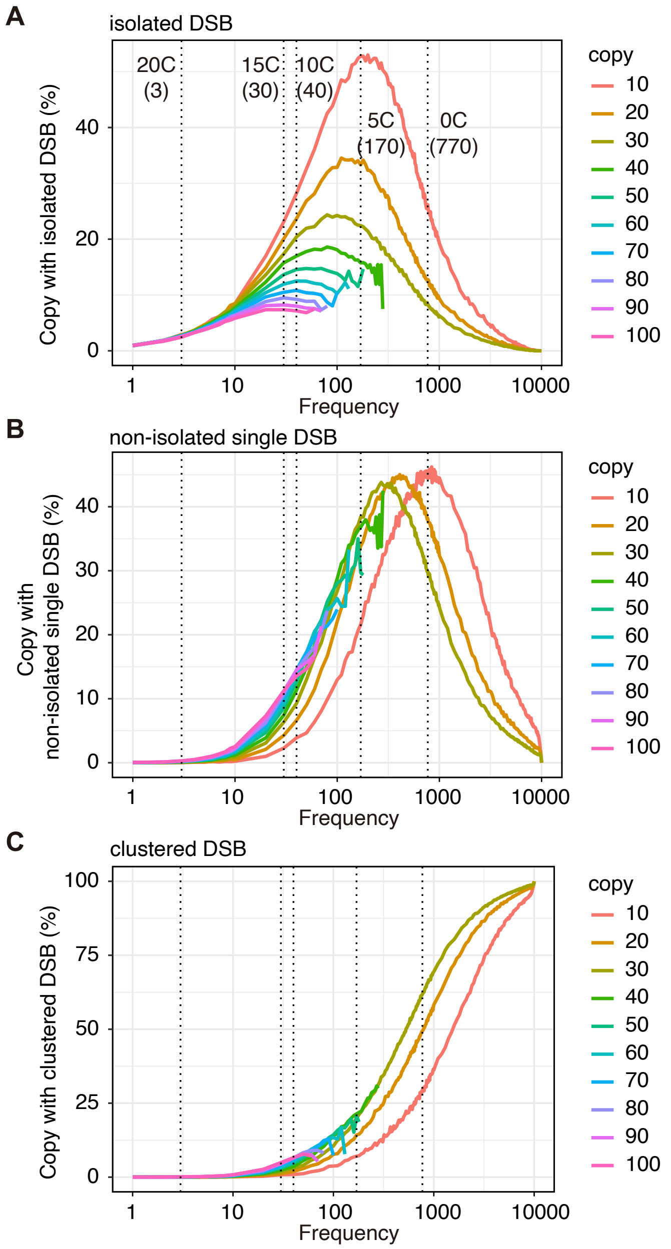

Supplementary Figure. S11: Dynamics of single vs multiple DSBs.

(A-C) Dynamics of the fractions of copies exhibiting temporally isolated single DSBs (A), temporally non-isolated single DSBs (B), or multiple DSBs (C) across varying Cas9 binding frequencies. This panel replots Fig. 6H separately for cells with different ecDNA copy numbers. The dotted lines represent the inferred frequencies of Cas9 binding for each sgRNA, and the corresponding sgRNAs are indicated in (A).

Supplementary Figure S12

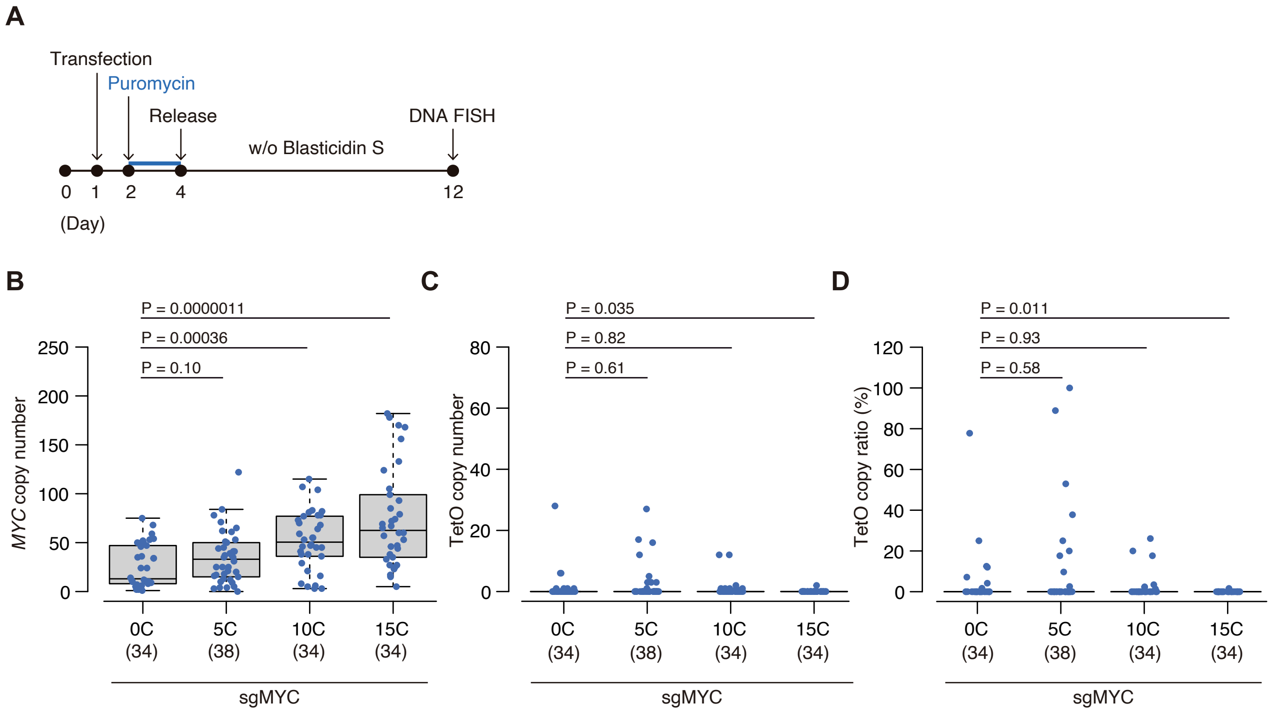

Supplementary Figure S12: Analysis of ecDNA copy number and knock-in efficiency without blasticidin S selection.

(A) Experimental scheme for DNA FISH analyses shown in (B–D).

(B, C) Box-and-whisker plots showing copy numbers of (B) MYC ecDNA and (C) TetO knock-in ecDNA in cells transfected with all-in-one CRISPR plasmids with the indicated sgRNAs and the knock-in template plasmid are shown.

(D) Box-and-whisker plots showing the fraction of MYC ecDNA carrying the TetO knock-in sequence.

Sample sizes (n) are shown below sgRNA labels. Statistical significance was assessed using a Wilcoxon rank-sum test.

Supplementary Figure S13

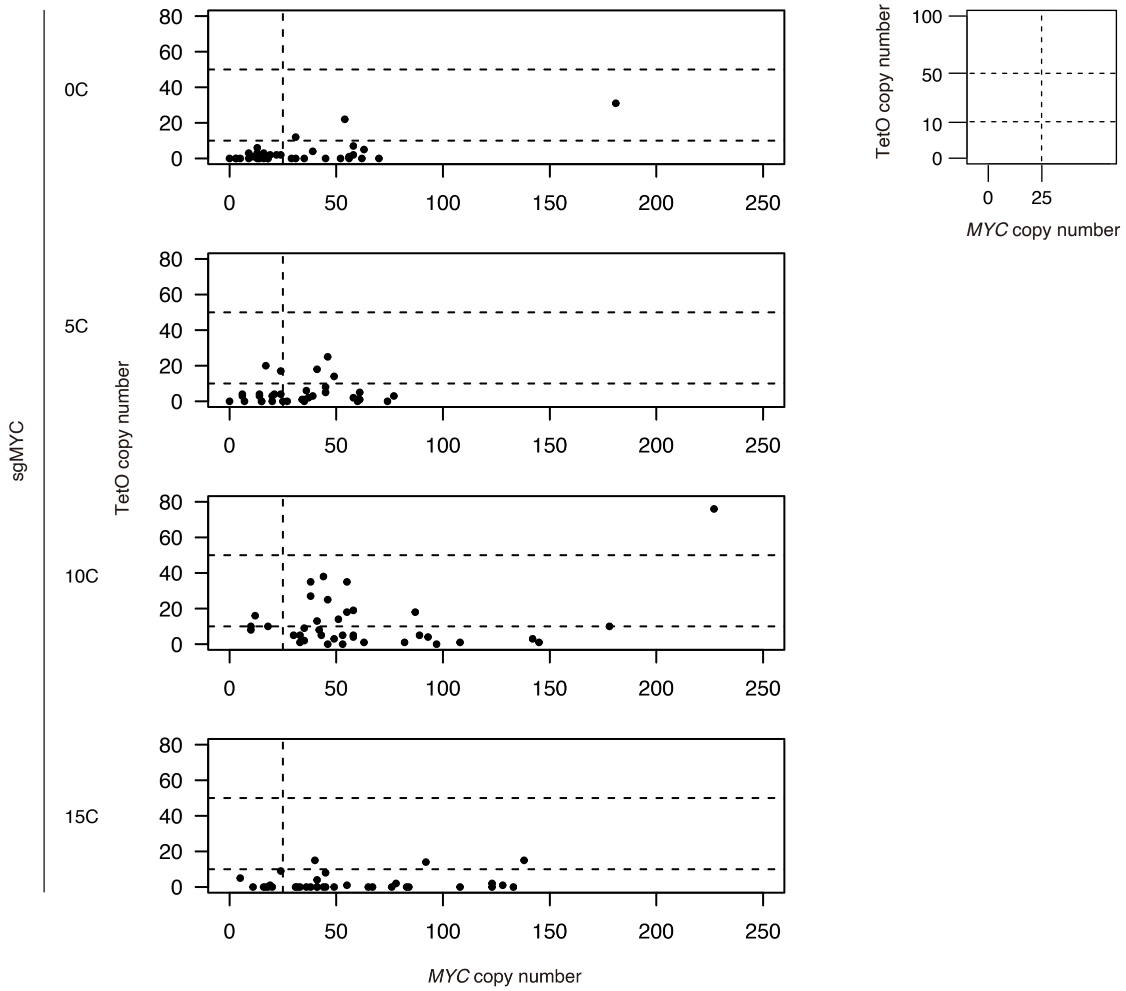

Supplementary Figure S13: Relationship between ecDNA copy number and TetO knock-in copy number.

Scatter plots of individual cells are shown.

Supplementary Figure S14

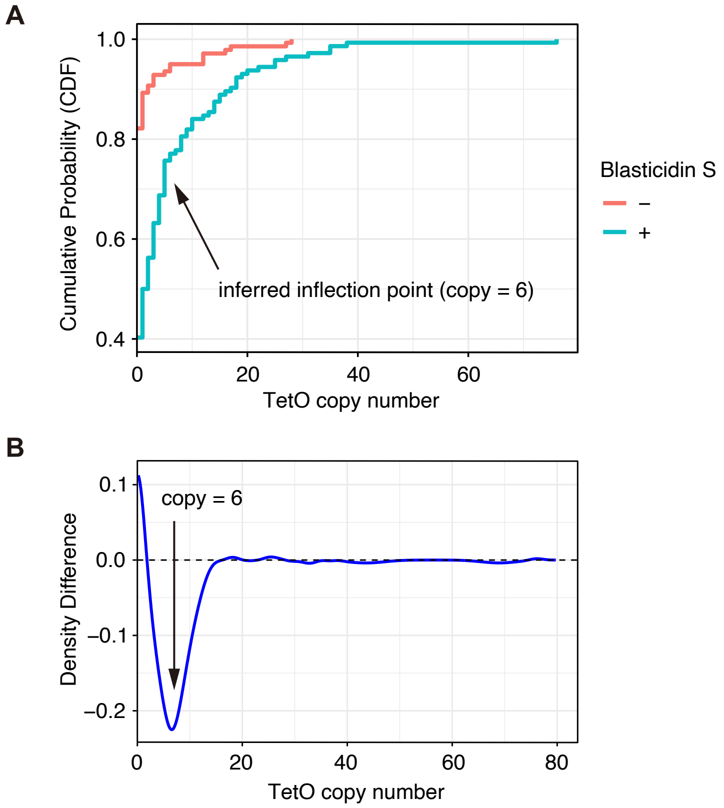

Supplementary Figure. S14: Comparison of TetO knock-in ecDNA copy distribution in the absence and presence of blasticidin S.

(A) CDF plot showing the distribution of TetO knock-in ecDNA copy number in the absence and presence of blasticidin S. The analysis suggested an inflection point at a TetO copy number of 6.

(B) Plot shows differences in density of TetO knock-in ecDNA copy distribution between the absence and presence of blasticidin S.

Supplementary Figure S15

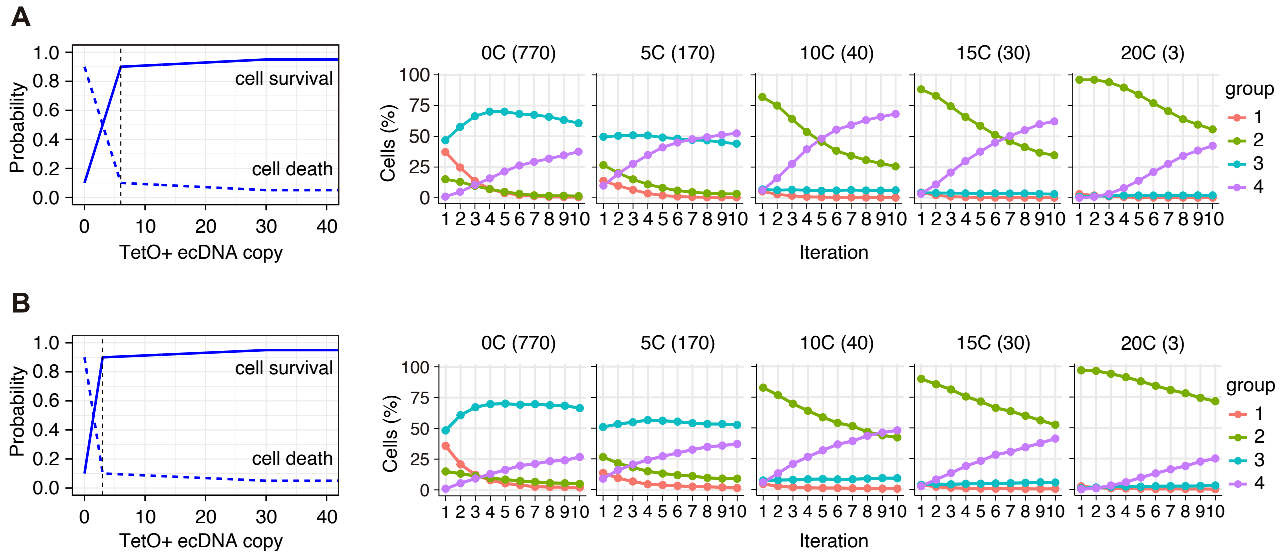

Supplementary Figure. S15: Simulations of cell dynamics with different selection schemes.

(A, B) Two examples of simulations. Left panels show scheme of blasticidin S selection. Right panels show cell dynamics under blasticidin S selection.
